# Prevalence of *Giardia duodenalis* among Asian children: a systematic review and meta-analysis

**DOI:** 10.1093/inthealth/ihad037

**Published:** 2023-05-19

**Authors:** Sara Kalavani, Sara Matin, Vahid Rahmanian, Ahmad Meshkin, Ali Taghipour, Amir Abdoli

**Affiliations:** Zoonoses Research Center, Jahrom University of Medical Sciences, Jahrom, Iran; Student Research Committee, Jahrom University of Medical Sciences, Jahrom, Iran; Department of Pediatrics, Jahrom University of Medical Sciences, Jahrom; Department of Public Health, Torbat Jam Faculty of Medical Sciences, Torbat Jam, Iran; Student Committee of Medical Education Development, Education Development Center, Gerash University of Medical Sciences, Gerash, Iran; Zoonoses Research Center, Jahrom University of Medical Sciences, Jahrom, Iran; Department of Medical Parasitology and Mycology, School of Medicine, Jahrom University of Medical Sciences, Jahrom, Iran; Zoonoses Research Center, Jahrom University of Medical Sciences, Jahrom, Iran; Department of Medical Parasitology and Mycology, School of Medicine, Jahrom University of Medical Sciences, Jahrom, Iran

**Keywords:** Asia, children, *Giardia duodenalis*, meta-analysis

## Abstract

*Giardia duodenalis* is one of the major causes of diarrhea among children. We performed a systematic review and meta-analysis to assess the prevalence of *G. duodenalis* and associated risk factors among Asian children. We searched online databases (PubMed, Scopus and Web of Science) and Google Scholar search engine for studies published from 1 January 2000 to 15 March 2022 that measured the prevalence of *G. duodenalis* among Asian children. Accordingly, the pooled prevalence and 95% CIs were estimated using a random-effects meta-analysis model for the included studies. A total of 182 articles from 22 Asian countries met the inclusion criteria. The pooled prevalence of *G. duodenalis* infection among Asian children was estimated as 15.1% (95% CI 14.1 to 16%). The highest and lowest pooled prevalence values of *G. duodenalis* infection were estimated for Tajikistan and China as 26.4% (95% CI 22.9 to 30%) and 0.6% (95% CI 0.001 to 1.02%), respectively. The infection had a higher prevalence in males than in females (OR=1.24; 95% CI 1.16 to 1.31; p<0.001), which was statistically significant. Giardiasis is common among Asian children, hence, a prevention and control scheme of this protozoan in children should be considered by health officials and health policymakers, especially in Asian countries where the prevalence is highest.

## Introduction

Diarrhea is one of the major causes of morbidity and mortality among children, particularly those living in underdeveloped or developing nations.^1–3^ Accordingly, unsafe water, sanitation and childhood malnutrition are the leading risk factors for diarrhea, especially in south Asia and sub-Saharan Africa.^1,4^ Overall, diarrhea is the cause of mortality of about 480 000 young children worldwide, and in 2019 was responsible for 9% of all deaths among children aged <5 y (https://data.unicef.org/topic/child-health/diarrhoeal-disease/). According to WHO reports in 2010, giardiasis is estimated to cause approximately 28.2 million cases of diarrhea.^5,6^ Among infectious agents, giardiasis is one of the major causes of diarrhea in developing countries.^7^  *Giardia duodenalis* is an intestinal protozoan parasite with a worldwide distribution in human and animals.^8–10^ The fecal–oral route is the main transmission mode of *G. duodenalis*.^8,10,11^ Drinking water, food and vegetables contaminated by *G. duodenalis* cysts are the main sources of transmission.^10,12,13^ The distribution of *G. duodenalis* in different communities is related to sanitation status, health measures and the quality of the drinking water.^14^ In this regard, the prevalence of *G. duodenalis* is much lower in developed countries than in less-developed geographical areas.^7,15^ Symptomatic giardiasis is significantly associated with worsening of nutritional status among children aged <5 y in Asian and African countries.^16^ The common clinical manifestations of giardiasis include greasy diarrhea (steatorrhea), nausea, vomiting, abdominal bloating, cramps, malabsorption and weight loss.^17,18^ Moreover, chronic giardiasis is related to food allergies,^19^ irritable bowel syndrome,^20^ chronic fatigue syndrome^21^ and arthritis,^22^ as well as growth deficiency in children.^23^ Previous studies have shown that individuals with a weak immune system, such as pregnant women and immunocompromised patients (e.g. with cancer, HIV/AIDS, etc.), are at a higher risk of *G. duodenalis* infection.^10,24–26^ Also, children are at a higher risk of *G. duodenalis* infection.^10,24–26^ Children have more contact with different environmental sources and also have immature immune systems to fight infections.^27,28^ Therefore, this group has a higher probability of contracting infectious agents. According to a recent meta-analysis,^29^ the risk factors for giardiasis were determined as exposure to sewage/wastewater, untreated drinking water and recreational waters. Interestingly, contact with pets was found to be a significant risk factor for giardiasis in children. Travel abroad was a risk factor for giardiasis in industrialized countries.^29^ A previous meta-analysis by Muhsen and Levine^7^ revealed the association between endemic pediatric diarrhea and giardiasis in developing countries. Asia is the world's largest continent with the highest human population. A large number of underdeveloped or developing countries are located in Asia. Therefore, determination of the epidemiological patterns of *G. duodenalis* infection in children is necessary to design future control programs and preventive measures to reduce the incidence of the infection in Asian countries. To address this gap, we designed a systematic review and meta-analysis to assess the prevalence of *G. duodenalis* and associated risk factors in children in Asia.

## Methods

### Information sources and systematic search

This systematic review and meta-analysis was followed based on the Preferred Reporting Items for Systematic Reviews and Meta-analyses (PRISMA) protocol.^30^ Published literature on the prevalence of *G. duodenalis* in children in Asia was retrieved through three major databases (i.e. PubMed, Scopus and Web of Science) and Google Scholar search engine from 1 January 2000 to 15 March 2022. The search process was accomplished using Medical Subject Headings (MeSH) terms alone or in combination: (‘Intestinal protozoa’ OR ‘*Giardia*’ OR ‘Giardiasis’) AND (‘Prevalence’ OR ‘Epidemiology’) AND (‘Children’). Moreover, the reference lists of all the selected articles were hand-searched to find other relevant articles or their citations by searching in Google Scholar.

### Inclusion criteria, study selection and data extraction

To assess article eligibility based on the determined inclusion criteria, all the papers were reviewed by two independent reviewers and possible contradictions among studies were removed by discussion and consensus. The inclusion criteria for this systematic review were: (1) full texts or abstracts published in English from Asia; (2) peer-reviewed original research papers or short reports; (3) cross-sectional studies that estimated the prevalence of *Giardia* in children (aged ≤18 y); (4) utilizing fecal microscopy, coproantigen or molecular diagnostic methods; (5) reports with information on the total sample size and positive samples; and (6) published online from 1 January 2000 to 15 March 2022. Those papers without full-text accessibility or papers that did not meet the above criteria were excluded. Next, the desired data were gathered precisely using a data extraction form including each first author's surname, the year in which the study was conducted and the publication year, countries, provinces or cities, the types of method used, total sample sizes, the number of positive samples, types of children, gender and age of the children, as well as the presence or absence of diarrhea.

### Study quality assessment

The Joanna Briggs Institute (JBI) checklist was applied for the risk of bias (internal validity) assessment of the included articles.^31^ This checklist comprises 10 questions, with four options consisting of Yes, No, Unclear and Not applicable. In summary, a study can be awarded a maximum of one star for each numbered item. Those papers with a total score of either 4–6 or 7–10 points were specified as being of moderate or high quality, respectively. Based on the score each paper obtained, the authors decided to either include (4–10 points) or exclude (≤3 points) it.

### Meta-analysis

For each of the included studies, the point estimates and their respective 95% CIs were calculated using a random effect model (REM). The REM allows for a distribution of true effect sizes between articles. To visualize possible heterogeneity among the included studies, forest plot analysis was used. The heterogeneity index among the included studies was defined using the *I*^2^ index and Tau squared to reveal the variation in study outcomes between individual studies.^32,33^ Univariate and multivariable meta-regression analysis was used to estimate the effects of probable factors in heterogeneity.^33^ To investigate the effect of each study on the pooled estimation of prevalence, the sensitivity analysis method was used by removing studies one by one. The robustness of each model was evaluated and, finally, the most favorable model was chosen.

Using subgroup analyses, the pooled prevalence of *Giardia* infection was estimated according to countries, types of diagnostic methods, types of children and the periods of studies. An OR (and the corresponding 95% CI) was calculated for each study to assess the association between *Giardia* spp. prevalence and risk factors such as sex (male and female) and place of living (rural and urban).

We used funnel plots and Egger's test for examining funnel plot asymmetry to assess the risk of bias due to missing results in a synthesis. Furthermore, the trim-and-fill method was used to estimate the number of censored studies and correct the overall estimate.^33^

Moreover, because of different sensitivities and specificities of diagnostic methods, we assumed that our results would be ‘apparent’ prevalence rates, which did not represent true prevalence rates. The prevalence of *G. duodenalis* in children in different countries of Asia was demonstrated as a world map using ArcGIS 10.3 software (https://www.arcgis.com). This meta-analysis was conducted with Stata version 16 software and the trial version of comprehensive meta-analysis software (version 3, BIOSTAT, Englewood, NJ, USA) version 3. p<0.05 was considered significant.

## Results

### Characteristics of the eligible studies

**Figure 1.**
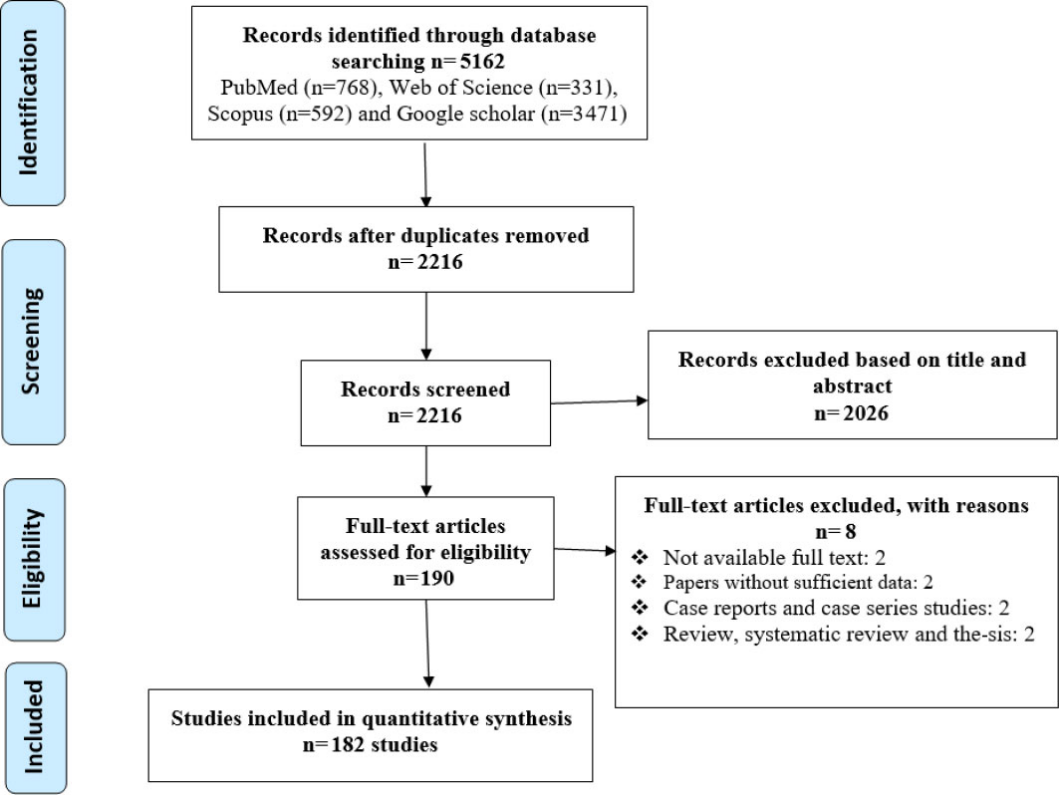
PRISMA flow diagram describing included/excluded studies.

A flowchart depicting the identification process of the qualifying studies is presented in Figure 1. In brief, the systematic search identified 5162 potentially relevant articles. After removing duplicates and/or non-eligible papers, 182 articles from 22 countries across Asia met the inclusion criteria in the systematic review and meta-analysis. The countries with the highest number of studies were Iran (22.52%; 41/182 studies) and Iraq (19.23%; 35/182 studies). The main characteristics of each study are shown in Supplementary Table 1. The results of quality assessment according to JBI with references for eligible studies are depicted in Supplementary Table 1. The articles included in the current meta-analysis displayed acceptable quality.

### The pooled prevalence of *G. duodenalis* in children

We estimated that the pooled prevalence of *G. duodenalis* for children in Asia, using REM, was 15.1% (95% CI 14.1 to 16%) (Q statistic=37713.61, d.f.=182, p<0.0001, *I*^2^=99.5%, Tau squared=0.0033); after sensitivity analysis using one-by-one removal of studies, the best robustness model was selected.

According to *I*^2^=99.5% and the significance of the Q statistic, it was detected that heterogeneity between studies was high, so univariate and multivariable meta-regression models were used to discover the causes of heterogeneity (Table 1). Univariate meta-regression analysis showed that year of study (p<0.001) could be the source of heterogeneity. In the next step, the multivariable meta-regression showed that year of study (B-coefficient= −0.00494, p<0.001) might have been the source of heterogeneity. This means that a change in the publication year can decrease the prevalence by −0.00494. Furthermore, the multivariable meta-regression analysis did show a significant difference in the location of studies, sample size, type of study population and year of study (p<0.05) (Table 1).

**Table 1.** Univariate and multivariable meta-regression to find possible causes of heterogeneity between studies included in the meta-analysis; N=183, tau^2^=0.004751, I-squared residual=99.09%, Adj R-squared= −43.63%, p=0.035

	Univariate		Multivariable	
Possible cause of heterogeneity	Coefficient (95% CI)	p	Coefficient (95% CI)	p
Location	−0.00023 (−0.00436 to 0.00389)	0.115	−0.00348 (−0.00378 to −0.00318)	**0.047**
Risk of bias	−0.01761 (−0.04501 to 0.00977)	0.206	−0.02034 (−0.04811 to 0.00742)	0.150
Year	−0.00431 (−0.00458 to −0.00405)	**<0.001**	−0.00494 (−0.00519 to −0.00470)	**<0.001**
Sample size	−1.1606 (−2.8906 to 5.7007)	0.189	−1.0907 (−1.5207 to −6.6808)	**<0.001**
Type of study population	−0.00185 (−0.00386 to 0.00016)	0.071	0.00145 (0.00130 to 0.00160)	**<0.001**
Diagnostic method	0.02832 (−0.00572 to 0.06238)	0.103	0.03422 (−0.00145 to 0.06990)	0.060

Significant difference with p<0.05.

**Figure 2.**
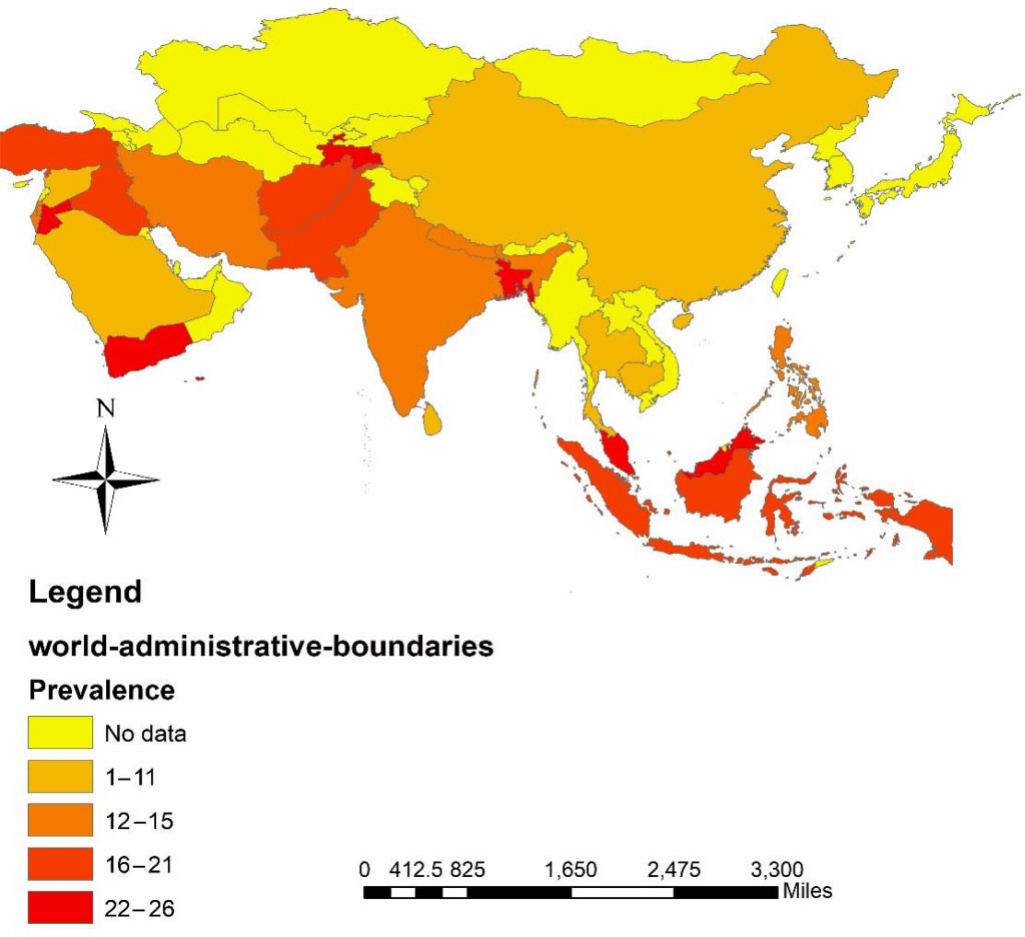
Prevalence of *G. duodenalis* in children based in countries in Asia.

Based on geographical regions, among the countries, the highest and lowest pooled prevalence values of *G. duodenalis* for children in Asia were estimated for Tajikistan and China as 26.4% (95% CI 22.9 to 30%) and 0.6% (95% CI 0.001 to 1.02%), respectively (Table 2). Also, schematic maps of the prevalence of *G. duodenalis* in children were created based on studies conducted in various countries in Asia (Figure 2). Based on the type of study population, the estimated *G. duodenalis* prevalence is: children with *Helicobacter pylori* 48.6% (95% CI 32.5 to 64.8%), preschool and school children (mixed) 42.1% (95% CI 19.4 to 78.3%), children with malignancies 22.6% (95% CI 13.4 to 31.7%), primary school children 17.8% (95% CI 12.4 to 23.3%), children (mixed) 16.5% (95% CI 15.4 to 17.6%), children living in a child care center 16% (95% CI 10.2 to 21.9%), preschool children 15% (95% CI 10.9 to 19.2%), school children 11.5% (95% CI 14.3 to 16%), children with diarrhea 10.7% (95% CI 7.08 to 13.5%), children with gastrointestinal disorders 6.07% (95% CI 2.02 to 11.3%) and malnourished children 0.08% (95% CI 0.01 to 14.9%) (Table 2). The pooled prevalence by type of diagnostic method is: based on ELISA 24.8% (95% CI 20.3 to 29.3%), microscopic examination 14.8% (95% CI 13.9 to 15.7%), PCR 35.2% (95% CI 21.1 to 49.2%) and real-time PCR assay 5.05% (95% CI 1.08 to 9.02%) (Table 2). In addition, the highest prevalence was in 2005 at 27.8% (95% CI 1.07 to 53.9%) and lowest in 2021 at 4.03% (95% CI 0.001 to 0.08%) and 2022 at 0.001% (95% CI 0.0001 to 0.002%) (Table 2).

**Table 2.** Pooled prevalence of *G. duodenalis* in children in Asia

						Heterogeneity			
Variables		No. of studies	No. examined	No. positive	Prevalence (95% CI)	χ^2^	p	*I*² (%)	Tau squared
Country	Afghanistan	1	245	52	21.2% (16.1 to 26.3%)	NA	NA	NA	NA
	Bangladesh	4	1429	414	24.5% (3.08 to 45.2%)	356.37	<0.001	99.2%	0.0441
	Cambodia	6	19 398	1599	8.09% (5.04 to 12.4%)	194.03	<0.001	97.4%	0.0018
	China	4	14 484	35	0.6% (0.001 to 1.02%)	18.55	<0.001	83.8%	0.00001
	India	19	7192	1131	15.3% (10.6 to 20%)	959.37	<0.001	98.1%	0.0106
	Indonesia	2	192	41	21.3% (6.03 to 36.4%)	6.81	0.009	85.3%	0.0100
	Iran	41	217 202	15 725	15.3% (13.8 to 16.8%)	5419.21	<0.001	99.2%	0.0021
	Iraq	35	25 338	4057	17.2% (14 to 20.3%)	4074.52	<0.001	99.2%	0.0086
	Israel	2	45 490	5228	11.8% (9.09 to 13.7%)	1.23	0.268	18.6%	0.0001
	Jordan	2	2665	1025	21.7% (19.4 to 62.7%)	1346.84	<0.001	99.9%	0.0876
	Malaysia	4	1082	276	26% (18.1 to 34%)	24.88	<0.001	87.9%	0.0055
	Nepal	14	11 422	2774	15.3% (5.01 to 25.6%)	4247.52	<0.001	99.7%	0.0377
	Pakistan	14	4112	669	18.4% (13.4 to 23.4%)	361.62	<0.001	96.4%	0.0083
	Palestine	5	5434	756	11.8% (5.02 to 18.4%)	266.54	<0.001	98.5%	0.0056
	Philippines	1	172	20	11.6% (6.08 to 16.4%)	NA	NA	NA	NA
	Saudi Arabia	7	2824	139	6.04% (3.07 to 9%)	125.48	<0.001	95.2%	0.0010
	Sri Lanka	2	345	33	9.01% (4.06 to 13.6%)	2.23	0.136	55.1%	0.0006
	Syria	3	2943	346	10.9% (5.07 to 16.1%)	42.66	<0.001	95.3%	0.0020
	Tajikistan	1	594	157	26.4% (22.9 to 30%)	NA	NA	NA	NA
	Thailand	10	6405	360	8.06% (6.02 to 11.1)	255.25	<0.001	96.5%	0.0013
	Turkey	1	357	67	18.8% (14.7 to 22.8%)	NA	NA	NA	NA
	Yemen	4	10 206	771	25.3% (6.09 to 43.6%)	454.46	<0.001	99.3%	0.0347
Type of study population	Children (mixed)	79	276 440	21 005	16.5% (15.4 to 17.6%)	20 366.69	<0.001	99.6%	0.0022
	Children living in the child care center	8	2396	382	16% (10.2 to 21.9%)	130.32	<0.001	94.6%	0.0064
	Children with diarrhea	13	8145	686	10.7% (7.08 to 13.5%)	495.11	<0.001	97.6%	0.0024
	Children with gastrointestinal disorders	4	1511	95	6.07% (2.02 to 11.3%)	50.15	<0.001	94.0%	0.0020
	Children with *Helicobacter pylori*	1	37	18	48.6% (32.5 to 64.8%)	NA	NA	NA	NA
	Children with malignancies	2	195	45	22.6% (13.4 to 31.7%)	2.49	0.114	59.9%	0.0026
	Malnourished children	2	185	16	0.08% (0.01 to 14.9%)	3.22	0.073	68.9%	0.0017
	Mixed of preschool and school children	2	2303	1369	42.1% (19.4 to 78.3%)	1329.19	<0.001	99.9%	0.1963
	Preschool children	11	3740	505	15% (10.9 to 19.2%)	164.14	<0.001	93.9%	0.0044
	Primary school children	17	12 775	2824	17.8% (12.4 to 23.3%)	1115.77	<0.001	98.6%	0.0128
	School children	43	71 804	8730	11.5% (14.3 to 16%)	3837.01	<0.001	98.9%	0.0047
Diagnostic method	ELISA	2	350	87	24.8% (20.3 to 29.3%)	0.27	0.605	0.0%	0.0000
	Microscopic examination	173	374 931	35 083	14.8% (13.9 to 15.7%)	36 808.84	<0.001	99.5%	0.0034
	PCR	4	1221	432	35.2% (21.1 to 49.2%)	80.29	<0.001	96.3%	0.0196
	Real-time PCR assay	3	3029	73	5.05% (1.08 to 9.02%)	37 713.61	<0.001	99.5%	0.0033
Year	2000	5	3850	772	18.3% (6.04 to 30.2%)	623.74	<0.001	99.4%	0.0181
	2001	4	4932	963	14.2% (6.04 to 12.2%)	130.95	<0.001	97.7%	0.0061
	2002	8	3090	357	12.8% (8.01 to 17.4%)	125.75	<0.001	94.4%	0.0040
	2003	8	10 684	750	20% (12.4 to 27.6%)	262.96	<0.001	97.3%	0.0111
	2004	12	71 199	6305	12.9% (7.08 to 18%)	1497.80	<0.001	99.3%	0.0079
	2005	6	3967	1681	27.8% (1.07 to 53.9%)	2020.27	<0.001	99.8%	0.1058
	2006	7	11 438	2272	18.6% (9.02 to 28.1%)	1786.97	<0.001	99.7%	0.0161
	2007	5	4437	746	19.5% (3.04 to 35.7%)	820.62	<0.001	99.5%	0.0335
	2008	10	6348	1290	20.4% (13.2 to 27.6%)	633.26	<0.001	98.6%	0.0130
	2009	6	5372	1232	27.4% (6.09 to 47.9%)	1776.52	<0.001	99.7%	0.0650
	2010	7	2648	416	15% (10.3 to 19.7%)	74.40	<0.001	91.9%	0.0037
	2011	16	132 764	7567	13.2% (10 to 16.4%)	1038.87	<0.001	98.6%	0.0039
	2012	9	21 112	1575	8.03% (5.05 to 11.2%)	393.21	<0.001	98.0%	0.0016
	2013	18	11 071	775	11.3% (8.05 to 14.1%)	733.40	<0.001	97.7%	0.0033
	2014	16	53 515	6381	15.2% (12.1 to 18.4%)	853.86	<0.001	98.1%	0.0039
	2015	7	4428	604	13.3% (7.03 to 19.3%)	273.97	<0.001	97.8%	0.0061
	2016	7	2105	474	19.7% (7.04 to 32.1%)	589.27	<0.001	99.0%	0.0271
	2017	4	1051	110	14.8% (3.02 to 26.4%)	102.65	<0.001	97.1%	0.0133
	2018	11	5854	781	14.4% (9.07 to 19%)	385.01	<0.001	97.4%	0.0058
	2019	6	4556	182	7.05% (4.05 to 10.5%)	163.12	<0.001	96.9%	0.0011
	2020	6	1826	356	23.3% (10 to 36.6%)	309.67	<0.001	98.4%	0.0268
	2021	3	1709	75	4.03% (0.001 to 0.08%)	49.34	<0.001	95.9%	0.0013
	2022	1	11 575	11	0.001% (0.0001 to 0.002%)	NA	NA	NA	NA

Abbreviation: NA, not applicable.

### Risk factors

**Figure 3.**
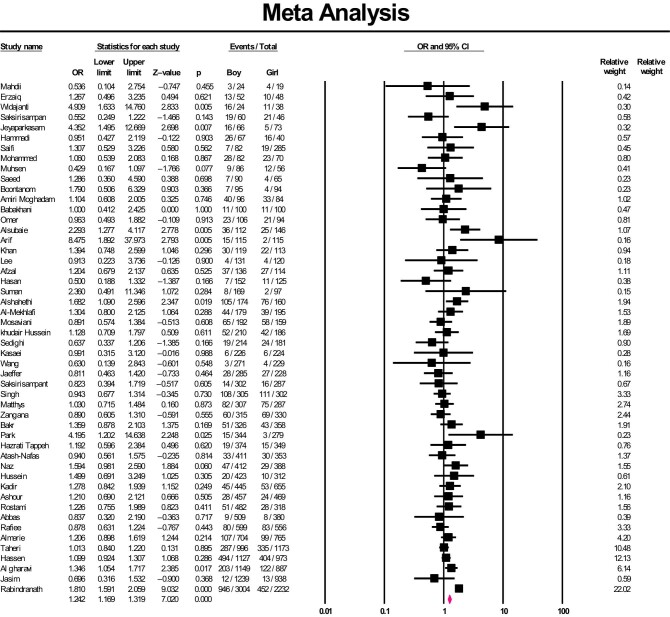
Association between male gender and prevalence of *G. duodenalis* in children.

**Figure 4.**
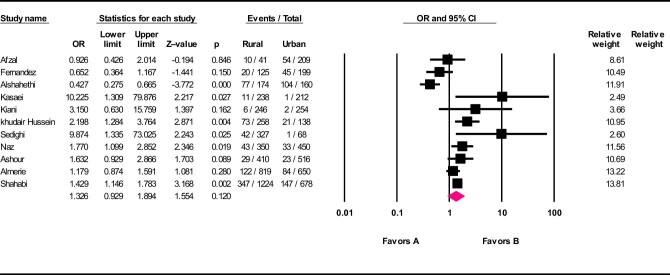
Association between living are and prevalence of *G. duodenalis* in children.

Based on children's gender, males had a higher prevalence of *G. duodenalis* than females (OR=1.24; 95% CI 1.16 to 1.31; p<0.001) (Q statistic=110.18, d.f.=49, p<0.0001, *I*^2^=55.53%, Tau squared=0.064), which was statistically significant (Figure 3). Moreover, children living in rural areas had a higher infection rate than those in urban areas (OR=1.32; 95% CI 0.92 to 1.89; p=0.120) (Q statistic=46.13, d.f.=10, p<0.0001, *I*^2^=78.32%, Tau squared=0.226); however, this was not statistically significant (Figure 4).

### Publication bias

**Figure 5.**
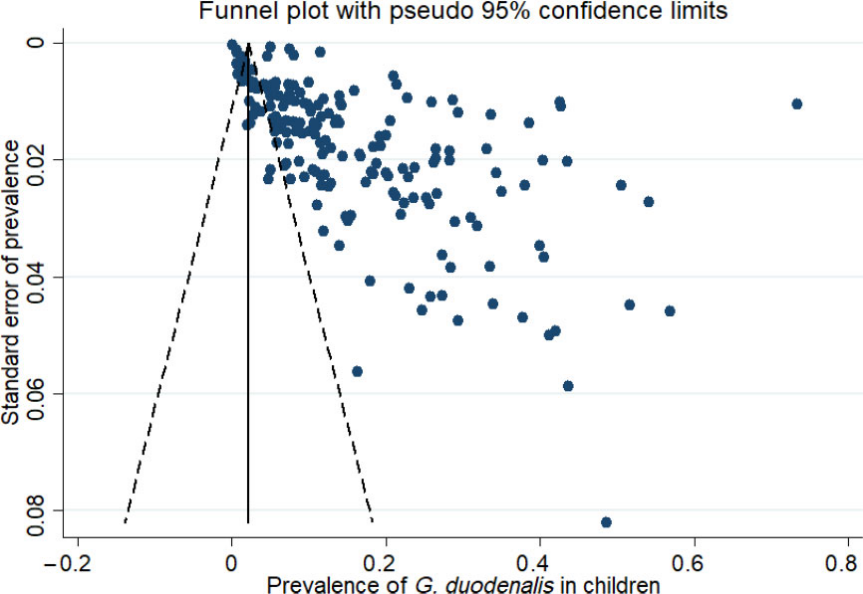
Funnel plot with pseudo 95% confidence limits for the detection of publication bias in this meta-analysis.

The funnel plots and Egger's test show that there is publication bias for studies reporting the prevalence of *G. duodenalis* in children (bias=9.22034, 95% CI 7.377599 to 11.0631; p<0.001) (Figure 5).

According to the trim-and-fill method, 90 hypothetical censored studies were estimated using non-parametric methods and regarded in the corrected meta-analysis. Therefore, the overall prevalence in children corrected by the REM based on the trim-and-fill model was estimated to be 3.04% (95% CI 2.05 to 4.03%) (Table 3).

**Table 3.** Comparison of common and corrected meta-analysis results for publication bias

Type of meta-analysis	Method	Number of studies	Pooled prevalence (95% CI)
Usual meta-analysis	Random-effects	182	15.1% (14.3 to 16%)
Filled meta-analysis	Random-effects	273	3.04% (2.05 to 4.03%)

## Discussion

This is the first study to estimate the prevalence of *G. duodenalis* among children in Asian countries. The prevalence values obtained for *G. duodenalis* in the meta-analysis were relatively high, especially in studies that used the PCR method (Table 2). Traditionally, the microscopy detection method using staining procedures is considered the gold standard for the detection of cysts and/or trophozoites of *G. duodenalis*.^34^ However, molecular methods are preferred for conducting research activities because they have higher sensitivity and specificity and interpretation of the results is easier.^35,36^ Accordingly, the pooled prevalence provided by molecular methods could be closer to the true prevalence. For a deeper understanding of this issue, it is necessary for researchers to use molecular methods in addition to microscopy methods.

When considering the prevalence of *G. duodenalis* in different Asian countries, the highest infection rates were found in Tajikistan (one study with a pooled prevalence of 26.4%) and Malaysia (four studies with a pooled prevalence of 26%). By contrast, infection rates in China (four studies with a pooled prevalence of 0.6%) and Saudi Arabia (seven studies with a pooled prevalence of 6.04%) were relatively low. Several environmental and sociodemographic parameters are complicated in the different prevalence rates obtained, including climatic condition, parasite control measures, the Human Development Index and the use of diverse diagnostic methods in different regions. In addition to all these factors, it is necessary to conduct more studies in Asian countries, and those countries that have not conducted any research on this issue should consider doing so; thus, a deeper understanding of the prevalence of *G. duodenalis* in children in different parts of Asia will be obtained.

Considering the year of publication (Table 2), during 2021 and 2022, the pooled prevalence of *G. duodenalis* infection decreased. This can be attributed to the increased health knowledge awareness of people.^37,38^ Furthermore, during the COVID-19 pandemic, because of enhanced personal and social hygiene, most food- and waterborne infectious diseases were relatively reduced.^39,40^ The results of this meta-analysis study, especially the pooled prevalence rates based on the year of publication, should be interpreted with caution, as a source of heterogeneity has been suggested by meta-regression analysis (Table 1). Factors such as the number of published articles and the sample size of studies each year may play a role in causing heterogeneity.

The current risk factor analyses showed that males and people living in rural areas are at a greater risk of exposure to *G. duodenalis*, which might be explained by lower personal hygiene scores and more contact with *G. duodenalis* cyst-contaminated water and vegetables.

The current study has a number of limitations. First, despite our comprehensive search, there was a paucity or absence of data for a number of countries, and many of the available studies had limited sample sizes and a lack of data on sociodemographic and/or risk factors. Moreover, in some countries, only one or two eligible studies were identified, which could compromise somewhat the interpretation of current estimates. Second, the studies included were undertaken during different time periods, with an absence of recent data for some countries, limiting the accuracy of inter-regional comparisons. Third, there was high heterogeneity in this meta-analysis, although we investigated its possible source by performing meta-regression analysis.

In summary, a prevention and control scheme of *G. duodenalis* in children should receive greater attention from health officials and health policymakers, especially in Asian countries where the prevalence is highest.

## Supplementary Material

ihad037_Supplemental_File

## Data Availability

All data during study are included in this manuscript and Supplementary Files.
