# Supplementary material for: Prevalence of Giardia duodenalis among Asian children: a systematic review and meta-analysis
Source: Int Health. 2023 May 19;16(2):133–43. doi: 10.1093/inthealth/ihad037 (PMC10911531; doi:10.1093/inthealth/ihad037)
Supplement: ihad037_Supplemental_File [file ihad037_supplemental_file.docx]

| **Supplementary Table 1.** Main characteristics of all eligible studies reporting prevalence of *Giardia duodenalis* in Asian children. | | | | | | | | | | |
| --- | --- | --- | --- | --- | --- | --- | --- | --- | --- | --- |
| **First author** | **Publication Year** | **Type of population** | **Age range or Mean age** | **Diagnostic method** | **Country** | **Continent** | **Sample size** | **Infected** | **QA** | **Reference** |
| Amiri Moghadam and Khansari | 2000 | Primary school children | NR | Microscopic examination | Iran | Asia | 180 | 73 | 8 | [1] |
| Youssef et al. | 2000 | Children under 5 years | 5 years | Microscopic examination | Jordan | Asia | 265 | 2 | 9 | [2] |
| Kalantari and Mobadi | 2000 | Children living in the Child Care Center | NR | Microscopic examination | Iran | Asia | 348 | 40 | 9 | [3] |
| Rafiee et al. | 2000 | Children 6-11 years | 6-11 years | Microscopic examination | Iran | Asia | 1155 | 163 | 10 | [4] |
| Shahabi | 2000 | School children | NR | Microscopic examination | Iran | Asia | 1902 | 494 | 10 | [5] |
| Arif and Ibrahim | 2001 | Orphans’ children | 1-15 years | Microscopic examination | Iraq | Asia | 230 | 17 | 9 | [6] |
| Saifi and Wajihullah | 2001 | School children | 5-13 years | Microscopic examination | India | Asia | 367 | 26 | 9 | [7] |
| Saidi and Sajadi | 2001 | School children | 6-15 years | Microscopic examination | Iran | Asia | 906 | 186 | 9 | [8] |
| Ghahramanlou et al. | 2001 | Primary school children | NR | Microscopic examination | Iran | Asia | 3429 | 734 | 10 | [9] |
| Mahdi and Ali | 2002 | Children living in the Child Care Center | Below 6 years | Microscopic examination | Iraq | Asia | 43 | 7 | 7 | [10] |
| Kaur et al. | 2002 | Children with diarrhea | 1-14 years | Microscopic examination | India | Asia | 127 | 14 | 8 | [11] |
| Lee et al. | 2002 | Primary school children | NR | Microscopic examination | Cambodia | Asia | 251 | 8 | 9 | [12] |
| Moghimi and Sharifi | 2002 | Preschool children | NR | Microscopic examination | Iran | Asia | 300 | 55 | 9 | [13] |
| Fernandez et al. | 2002 | School children | NR | Microscopic examination | India | Asia | 324 | 65 | 9 | [14] |
| Davami et al. | 2002 | Children | 1-13 years | Microscopic examination | Iran | Asia | 385 | 64 | 9 | [15] |
| Al-Hindi | 2002 | School children | 6-11 years | Microscopic examination | Palestine | Asia | 650 | 90 | 9 | [16] |
| Waikagul et al. | 2002 | School children | NR | Microscopic examination | Thailand | Asia | 1010 | 54 | 10 | [17] |
| Waqar et al. | 2003 | Children | Under 15 years | Microscopic examination | Pakistan | Asia | 89 | 22 | 7 | [18] |
| Widajanti et al. | 2003 | Children living in the Child Care Center | ≤5 years | Microscopic examination | Indonesia | Asia | 92 | 27 | 7 | [19] |
| Saksirisampant et al. | 2003 | Preschool children | Between 10 to 81 months | Microscopic examination | Thailand | Asia | 106 | 40 | 8 | [20] |
| Monawar Hosain et al. | 2003 | Primary school children | Between 5 and 13 years | Microscopic examination | Bangladesh | Asia | 149 | 23 | 8 | [21] |
| Ahmad-Rajabi et al. | 2003 | Children living in the Child Care Center | ≤7 years | Microscopic examination | Iran | Asia | 370 | 62 | 9 | [22] |
| Piangjai et al. | 2003 | School children | NR | Microscopic examination | Thailand | Asia | 403 | 41 | 9 | [23] |
| Heidari and Rokni | 2003 | Children living in the Child Care Center | <6 years | Microscopic examination | Iran | Asia | 461 | 121 | 9 | [24] |
| Azazy and Rajaa | 2003 | Children | 0-14 years | Microscopic examination | Yemen | Asia | 9014 | 414 | 10 | [25] |
| Chandrasena et al. | 2004 | Primary school children | 6-15 years | Microscopic examination | Sri Lanka | Asia | 145 | 10 | 8 | [26] |
| Baldo et al. | 2004 | Children | 1->15 years | Microscopic examination | Philippines | Asia | 172 | 20 | 8 | [27] |
| Zakai | 2004 | Primary school children | 6-14 years | Microscopic examination | Saudi Arabia | Asia | 231 | 13 | 9 | [28] |
| Chaudhry et al. | 2004 | Children | Less than 15 years | Microscopic examination | Pakistan | Asia | 287 | 34 | 9 | [29] |
| Rai et al. | 2004 | Children | 1-15 years | Microscopic examination | Nepal | Asia | 301 | 36 | 9 | [30] |
| Taheri et al. | 2004 | Children | NR | Microscopic examination | Iran | Asia | 399 | 152 | 9 | [31] |
| Sharma et al. | 2004 | School children | 4-19 years | Microscopic examination | Nepal | Asia | 533 | 36 | 9 | [32] |
| Saksirisampant et al. | 2004 | School children | 6-19 years | Microscopic examination | Thailand | Asia | 542 | 12 | 9 | [33] |
| Park et al. | 2004 | School children | NR | Microscopic examination | Cambodia | Asia | 623 | 18 | 9 | [34] |
| Astal | 2004 | Children | 6-11 years | Microscopic examination | Palestine | Asia | 1370 | 110 | 10 | [35] |
| Adnan Mohamad and Samih Sami | 2004 | Primary school children | 6-12 years | Microscopic examination | Jordan | Asia | 2400 | 1023 | 10 | [36] |
| Ashtiani et al. | 2004 | Children | NR | Microscopic examination | Iran | Asia | 64196 | 4841 | 10 | [37] |
| Chandrasena et al. | 2005 | Children | 2-15 years | Microscopic examination | Sri Lanka | Asia | 200 | 23 | 9 | [38] |
| Wadood et al. | 2005 | Children | 0-13 years | Microscopic examination | Pakistan | Asia | 220 | 70 | 9 | [39] |
| Sadjjadi and Tanideh | 2005 | Preschool children | 3-6 years | Microscopic examination | Iran | Asia | 337 | 77 | 9 | [40] |
| Basima | 2005 | Children | Less than 5 -14 years | Microscopic examination | Iraq | Asia | 350 | 45 | 9 | [41] |
| Daryani and Etehad | 2005 | Primary school children | 7-13 years | Microscopic examination | Iran | Asia | 1070 | 152 | 10 | [42] |
| Easow et al. | 2005 | Preschool and school children | NR | Microscopic examination | Nepal | Asia | 1790 | 1314 | 10 | [43] |
| Mosaviani | 2006 | Children | 1-6 years | Microscopic examination | Iran | Asia | 351 | 123 | 9 | [44] |
| Atash-Nafas et al. | 2006 | Primary school children | NR | Microscopic examination | Iran | Asia | 764 | 63 | 9 | [45] |
| Chhakda et al. | 2006 | School children | NR | Microscopic examination | Cambodia | Asia | 789 | 33 | 9 | [46] |
| Saksirisampant et al. | 2006 | School children | 3-12 years | Microscopic examination | Thailand | Asia | 1037 | 13 | 10 | [47] |
| Al-Saeed and Issa | 2006 | Children | 3 months-12 years | Microscopic examination | Iraq | Asia | 1261 | 486 | 10 | [48] |
| Al Jarousha | 2006 | School children | 6-15 years | Microscopic examination | Palestine | Asia | 2000 | 456 | 10 | [49] |
| Rabindranath et al. | 2006 | Children | 1-14 years | Microscopic examination | Nepal | Asia | 5236 | 1098 | 10 | [50] |
| Mohseni et al. | 2007 | Children living in the Child Care Center | Mean age of 5.33 +/- 0.96 | Microscopic examination | Iran | Asia | 252 | 44 | 9 | [51] |
| Aminzadeh et al. | 2007 | Primary school children | NR | Microscopic examination | Iran | Asia | 293 | 78 | 9 | [52] |
| Wongstitwilairoong et al. | 2007 | Preschool children | 3 months-5 years | Microscopic examination | Thailand | Asia | 472 | 87 | 9 | [53] |
| Ebadi et al. | 2007 | Children | 0-14 years | Microscopic examination | Iran | Asia | 1500 | 504 | 10 | [54] |
| Ngrenngarmlert et al. | 2007 | School children | 7-12 years | Microscopic examination | Thailand | Asia | 1920 | 33 | 10 | [55] |
| Mehraj et al. | 2008 | Children | 1-5 years | Microscopic examination | Pakistan | Asia | 218 | 63 | 9 | [56] |
| Younas et al. | 2008 | Children | 4-14 years | Microscopic examination | Pakistan | Asia | 239 | 74 | 9 | [57] |
| AL-Rubaee | 2008 | Primary school children | 6-11 years | Microscopic examination | Iraq | Asia | 252 | 53 | 9 | [58] |
| Davami et al. | 2008 | Children | 7-15 years | Microscopic examination | Iran | Asia | 410 | 33 | 9 | [59] |
| Al-Braiken | 2008 | Children | 0-10 years | Microscopic examination | Saudi Arabia | Asia | 500 | 16 | 9 | [60] |
| Falahi et al. | 2008 | Primary school children | 6-12 years | Microscopic examination | Iran | Asia | 500 | 97 | 9 | [61] |
| Mahfoth et al. | 2008 | Children | NR | Microscopic examination | Iraq | Asia | 600 | 242 | 9 | [62] |
| Al-Hindi and El-Kichaoi | 2008 | Preschool children | <10 months-5 years | Microscopic examination | Palestine | Asia | 679 | 70 | 9 | [63] |
| Almerie et al. | 2008 | Primary school children | 6-12 years | Microscopic examination | Syria | Asia | 1469 | 206 | 10 | [64] |
| Ali Mohammed | 2008 | Children | 1-13 years | Microscopic examination | Iraq | Asia | 1481 | 436 | 10 | [65] |
| Yusof et al. | 2009 | Children | NR | Microscopic examination | Malaysia | Asia | 71 | 31 | 7 | [66] |
| Mumtaz et al. | 2009 | Children | under 5 years | Microscopic examination | Pakistan | Asia | 269 | 68 | 9 | [67] |
| Shrestha et al. | 2009 | School children | NR | Microscopic examination | Nepal | Asia | 303 | 55 | 9 | [68] |
| Ajjampur et al. | 2009 | Children | NR | Microscopic examination | India | Asia | 452 | 155 | 9 | [69] |
| Hassen | 2009 | Children | 0-10 years | Microscopic examination | Iraq | Asia | 2100 | 898 | 10 | [70] |
| Jasim and Al-Mugdadi | 2009 | Children | NR | Microscopic examination | Iraq | Asia | 2177 | 25 | 10 | [71] |
| Gyawali et al. | 2010 | Primary school children | 4-10 years | Microscopic examination | Nepal | Asia | 182 | 23 | 8 | [72] |
| Rayan et al. | 2010 | School children | NR | Microscopic examination | India | Asia | 195 | 17 | 8 | [73] |
| Khan et al. | 2010 | Children | 0-14 years | Microscopic examination | Pakistan | Asia | 232 | 52 | 9 | [74] |
| Hasan | 2010 | Children | 2-4 years | Microscopic examination | Iraq | Asia | 277 | 18 | 9 | [75] |
| khudair Hussein | 2010 | Children with diarrhea | 0-10 years | Microscopic examination | Iraq | Asia | 396 | 94 | 9 | [76] |
| Al-Haddad and Baswaid | 2010 | Children | 6-13 years | Microscopic examination | Yemen | Asia | 600 | 115 | 9 | [77] |
| Mohammad Taher et al. | 2010 | Primary school children | NR | Microscopic examination | Syria | Asia | 766 | 97 | 9 | [78] |
| Al-Mohammed | 2011 | Children | 6-12 years | Microscopic examination | Saudi Arabia | Asia | 97 | 40 | 7 | [79] |
| Ajjampur et al. | 2011 | Children | NR | Microscopic examination | India | Asia | 116 | 66 | 8 | [80] |
| Boontanom et al. | 2011 | Preschool children | 3->6 years | Microscopic examination | Thailand | Asia | 189 | 11 | 8 | [81] |
| Suman et al. | 2011 | Children | < 6 months-5 years | Microscopic examination | Bangladesh | Asia | 266 | 10 | 9 | [82] |
| Rashid et al. | 2011 | School children | NR | Microscopic examination | India | Asia | 320 | 20 | 9 | [83] |
| Bisht et al. | 2011 | Children | 0-14 years | Microscopic examination | India | Asia | 335 | 19 | 9 | [84] |
| Kadir and Mohammad-Ali | 2011 | Children | 2-13 years | Microscopic examination | Iraq | Asia | 348 | 32 | 9 | [85] |
| Nazal et al. | 2011 | Children | 1 month- 10 years | Microscopic examination | Iraq | Asia | 495 | 55 | 9 | [86] |
| Jaeffer | 2011 | Preschool and school children | 0-12 years | Microscopic examination | Iraq | Asia | 513 | 55 | 9 | [87] |
| Matthys et al. | 2011 | Primary school children | 7-11 years | Microscopic examination | Tajikistan | Asia | 594 | 157 | 9 | [88] |
| Aher and Kulkarni | 2011 | School children | 6-12 years | Microscopic examination | India | Asia | 624 | 84 | 9 | [89] |
| Al Kafri and Ismail | 2011 | School children | NR | Microscopic examination | Syria | Asia | 708 | 43 | 9 | [90] |
| Hussein | 2011 | School children | 7-13 years | Microscopic examination | Palestine | Asia | 735 | 30 | 9 | [91] |
| Abbas et al. | 2011 | Children with diarrhea | 1-12 years | Microscopic examination | Saudi Arabia | Asia | 889 | 17 | 9 | [92] |
| Taheri et al. | 2011 | School children | 6-11 years | Microscopic examination | Iran | Asia | 2169 | 622 | 10 | [93] |
| Ashtiani et al. | 2011 | Children | Under 13 years | Microscopic examination | Iran | Asia | 124366 | 6306 | 10 | [94] |
| Khan et al. | 2012 | Children | 5-10 years | Microscopic examination | Pakistan | Asia | 100 | 2 | 8 | [95] |
| Hammadi | 2012 | Children | 6-12 years | Microscopic examination | Iraq | Asia | 112 | 38 | 8 | [96] |
| Baig et al. | 2012 | Children | NR | Microscopic examination | Pakistan | Asia | 150 | 41 | 8 | [97] |
| Shrestha et al. | 2012 | School children | ≤4-10 years | Microscopic examination | Nepal | Asia | 260 | 15 | 9 | [98] |
| Saksirisampant et al. | 2012 | Children | 1->12 years | Microscopic examination | Thailand | Asia | 589 | 30 | 9 | [99] |
| Rostami et al. | 2012 | School children | 8-12 years | Microscopic examination | Iran | Asia | 800 | 79 | 9 | [100] |
| Tiwary et al. | 2012 | Children | NR | Microscopic examination | India | Asia | 1209 | 32 | 10 | [101] |
| Qays Ibrahim | 2012 | Children | 1 month-12 years | Microscopic examination | Iraq | Asia | 1520 | 27 | 10 | [102] |
| Moore et al. | 2012 | Children | 0-16 years | Microscopic examination | Cambodia | Asia | 16372 | 1311 | 10 | [103] |
| Khavari and Rahimkhani | 2013 | Malnourished children | 6 month-5 years | Microscopic examination | Iran | Asia | 84 | 4 | 7 | [104] |
| Zabolinejad et al. | 2013 | Children with lymphohematopoietic malignancy | 1-18 years | Microscopic examination | Iran | Asia | 89 | 16 | 7 | [105] |
| Khadka et al. | 2013 | School children | 3-15 years | Microscopic examination | Nepal | Asia | 100 | 5 | 8 | [106] |
| Maharjan et al. | 2013 | kindergarten | NR | Microscopic examination | Nepal | Asia | 101 | 26 | 8 | [107] |
| Prownebon et al. | 2013 | Children | Less than 6 years | Microscopic examination | Thailand | Asia | 137 | 39 | 8 | [108] |
| Pradhan et al. | 2013 | School children | NR | Microscopic examination | Nepal | Asia | 194 | 25 | 8 | [109] |
| Sah et al. | 2013 | School children | 12-15 years | Microscopic examination | Nepal | Asia | 200 | 21 | 9 | [110] |
| Ashok et al. | 2013 | School children | 8.8 ± 2.11 years | Microscopic examination | India | Asia | 208 | 25 | 9 | [111] |
| Patel et al. | 2013 | Children with diarrhea | <1-12 years | Microscopic examination | India | Asia | 298 | 16 | 9 | [112] |
| Salman and Mussttafa | 2013 | Children | NR | Microscopic examination | Iraq | Asia | 310 | 63 | 9 | [113] |
| AlAyed et al. | 2013 | Children | < 5 years | Microscopic examination | Saudi Arabia | Asia | 326 | 3 | 9 | [114] |
| Al-Mekhlafi et al. | 2013 | School children | 7-12 years | Microscopic examination | Malaysia | Asia | 374 | 83 | 9 | [115] |
| Momen Heravi et al. | 2013 | School children | 6-16 years | Microscopic examination | Iran | Asia | 430 | 38 | 9 | [116] |
| Tiwari et al. | 2013 | School children | 4-12 years | Microscopic examination | Nepal | Asia | 530 | 13 | 9 | [117] |
| Zangana et al. | 2013 | School children | 6-15 years | Microscopic examination | Iraq | Asia | 645 | 129 | 9 | [118] |
| Mero and Hussein | 2013 | Children | 5 months-13 years | Microscopic examination | Iraq | Asia | 1132 | 135 | 10 | [119] |
| Tandukar et al. | 2013 | School children | 0-15 years | Microscopic examination | Nepal | Asia | 1392 | 103 | 10 | [120] |
| Asl and Saei | 2013 | Children | NR | Microscopic examination | Iran | Asia | 4521 | 31 | 10 | [121] |
| Kazemian et al. | 2014 | Children with Helicobacter pylori | NR | Microscopic examination | Iran | Asia | 37 | 18 | 7 | [122] |
| Torabi et al. | 2014 | Children | 2-12 years | Microscopic examination | Iran | Asia | 124 | 64 | 8 | [123] |
| Muhsen et al. | 2014 | Preschool children | NR | Microscopic examination | Israel | Asia | 142 | 21 | 8 | [124] |
| Saeed and Khairi | 2014 | Children | Less than one year-12 years | Microscopic examination | Iraq | Asia | 155 | 11 | 8 | [125] |
| Anvari Tafti et al. | 2014 | Children living in the Child Care Center | NR | Microscopic examination | Iran | Asia | 180 | 5 | 8 | [126] |
| Ullah et al. | 2014 | School children | 4-15 years | Microscopic examination | Pakistan | Asia | 222 | 5 | 9 | [127] |
| Mane et al. | 2014 | Preschool children | 1-5 years | Microscopic examination | India | Asia | 385 | 47 | 9 | [128] |
| Al-Delaimy et al. | 2014 | School children | NR | Microscopic examination | Malaysia | Asia | 498 | 141 | 9 | [129] |
| Ahmed et al. | 2014 | Children | NR | Microscopic examination | Bangladesh | Asia | 591 | 167 | 9 | [130] |
| Kiran et al. | 2014 | School children | 6-12 years | Microscopic examination | India | Asia | 600 | 261 | 9 | [131] |
| Abdi et al. | 2014 | Children living in the Child Care Center | Under the age of 6 years | Microscopic examination | Iran | Asia | 650 | 76 | 9 | [132] |
| Hazrati Tappeh et al. | 2014 | Children | NR | Microscopic examination | Iran | Asia | 720 | 34 | 9 | [133] |
| Haji aliani et al. | 2014 | kindergarten | 1-6 years | Microscopic examination | Iran | Asia | 904 | 68 | 9 | [134] |
| Hamza and Al-Ibrahimi | 2014 | Children with diarrhea | ≤1-12 years | Microscopic examination | Iraq | Asia | 926 | 52 | 9 | [135] |
| AL Waqar et al. | 2014 | Children with diarrhea | 0-15 years | Microscopic examination | Iraq | Asia | 2033 | 204 | 10 | [136] |
| Ben-Shimol et al. | 2014 | School children | <5–19 years | Microscopic examination | Israel | Asia | 45348 | 5207 | 10 | [137] |
| Yasmeen and Singh | 2015 | Children | 3-15 years | Microscopic examination | India | Asia | 100 | 5 | 8 | [138] |
| Hussein et al. | 2015 | Children with diarrhea | 1 month - 18 years | PCR | Iraq | Asia | 100 | 42 | 8 | [139] |
| Pandey et al. | 2015 | School children | NR | Microscopic examination | Nepal | Asia | 300 | 4 | 9 | [140] |
| Sedighi et al. | 2015 | Children under 10 years | <10 years | Microscopic examination | Iran | Asia | 395 | 43 | 9 | [141] |
| Faraji et al. | 2015 | Children 0-6 y | 0-6 years | Microscopic examination | Iran | Asia | 632 | 88 | 9 | [142] |
| Moore et al. | 2015 | Children | 0-16 years | Microscopic examination | Cambodia | Asia | 865 | 97 | 9 | [143] |
| Al gharavi | 2015 | Children | <12 years | Microscopic examination | Iraq | Asia | 2036 | 325 | 10 | [144] |
| Erzaiq | 2016 | Children with malnutrition | 2m-5 years | Microscopic examination | Iraq | Asia | 101 | 12 | 8 | [145] |
| Yu et al. | 2016 | Kindergarten Children | NR | Microscopic examination | China | Asia | 125 | 3 | 8 | [146] |
| Omer and Alanazi | 2016 | Children | 9-12 years | PCR | Saudi Arabia | Asia | 200 | 44 | 9 | [147] |
| Alsubaie et al. | 2016 | School Children | 8-15 years | Microscopic examination | Yemen | Asia | 258 | 61 | 9 | [148] |
| Ahmed et al. | 2016 | Children | 13m-5 years | PCR | Bangladesh | Asia | 423 | 214 | 9 | [149] |
| Moore et al. | 2016 | Symptomatic Children | <16 years | PCR | Cambodia | Asia | 498 | 132 | 9 | [150] |
| Kiani et al. | 2016 | Children with gastrointestinal disorders | ≤ 12 years | Microscopic examination | Iran | Asia | 500 | 8 | 9 | [151] |
| Abdul-Hussein | 2017 | Children with malignancies | 2m-14 years | Microscopic examination | Iraq | Asia | 106 | 29 | 8 | [152] |
| Babakhani et al. | 2017 | School Children | 7-14 years | Microscopic examination | Iran | Asia | 200 | 22 | 9 | [153] |
| Lass et al. | 2017 | Children | 7-18 years | Real time PCR assay | Afghanistan | Asia | 245 | 52 | 9 | [154] |
| Wang et al. | 2017 | Diarrheal Children | <2 and >5 | Real time PCR assay | China | Asia | 500 | 7 | 9 | [155] |
| Effati et al. | 2018 | Children | 2-10 years | Microscopic/PCR | Iran | Asia | 160 | 15 | 8 | [156] |
| Al-Abodi | 2018 | Children | 0-2 years | Microscopic examination | Iraq | Asia | 200 | 80 | 9 | [157] |
| Qayyum et al. | 2018 | 5-12 years old Children with recurrent Abdominal pain | 5-12 years | Microscopic examination | Pakistan | Asia | 328 | 47 | 9 | [158] |
| Babat et al. | 2018 | Children 4-12 years | 4-12 years | Microscopic examination | Turkey | Asia | 357 | 67 | 9 | [159] |
| Chanu et al. | 2018 | Children with GE symptoms | 0-15 years | Microscopic examination | India | Asia | 400 | 20 | 9 | [160] |
| Kasaei et al. | 2018 | Children | 1-7 years | Microscopic examination | Iran | Asia | 450 | 12 | 9 | [161] |
| Singh et al. | 2018 | Children under 12 years | 1-12 years | Microscopic examination | India | Asia | 667 | 221 | 9 | [162] |
| Bakr et al. | 2018 | Local and Displaced Children | 1-13 years | Microscopic/ELISA | Iraq | Asia | 684 | 94 | 9 | [163] |
| Hafeez et al. | 2018 | Children | 1-14 years | Microscopic examination | Pakistan | Asia | 708 | 51 | 9 | [164] |
| Naz et al. | 2018 | Children | 0-10 years | Microscopic/ELISA | Pakistan | Asia | 800 | 76 | 9 | [165] |
| Kadir et al. | 2018 | Children with diarrhea | 6-12 years | Microscopic examination | Iraq | Asia | 1100 | 98 | 10 | [166] |
| Rather and Hussain Rather | 2019 | School-going children | 5-16 years | Microscopic examination | Indian | Asia | 130 | 10 | 8 | [167] |
| Jeyaparkasam and Ghani | 2019 | School Children | 6-13 years | Microscopic examination | Malaysia | Asia | 139 | 21 | 8 | [168] |
| Afzal et al. | 2019 | Children with acute watery diarrhea | 6m-12 years | ELISA | Pakistan | Asia | 250 | 64 | 9 | [169] |
| Bakarman et al. | 2019 | School Children | 6-16 years | Microscopic examination | Saudi Arabia | Asia | 581 | 6 | 9 | [170] |
| Hussein and Meerkhan | 2019 | Children in Hivi perdiatric Hospital | 1m-12 years | Microscopic examination | Iraq | Asia | 1172 | 67 | 10 | [171] |
| Yu et al. | 2019 | Hospitalized Children | <14 years | Real time PCR assay | China | Asia | 2284 | 14 | 10 | [172] |
| Erzaiq | 2020 | Children with persistant Diarrhea | 6m-5 years | ELISA | Iraq | Asia | 100 | 23 | 8 | [173] |
| Bachtiar et al. | 2020 | Elementary School Students | students in grade of 1st to 6^th^ | Microscopic examination | Indonesia | Asia | 100 | 14 | 8 | [174] |
| Mohammed et al. | 2020 | Children | 6m-5 years | Microscopic examination | Iraq | Asia | 152 | 51 | 8 | [175] |
| Alshahethi et al. | 2020 | Children | 1-14 years | Microscopic examination | Yemen | Asia | 334 | 181 | 9 | [176] |
| Teja et al. | 2020 | School Children | 6-14 years | Microscopic examination | India | Asia | 335 | 28 | 9 | [177] |
| Abed | 2020 | Children | 1-10 years | Microscopic examination | Iraq | Asia | 805 | 59 | 9 | [178] |
| Barati et al. | 2021 | Children with gastrointestinal disorders | <15 years | Microscopic examination | Iran | Asia | 283 | 20 | 9 | [179] |
| Matar et al. | 2021 | Children with diarrhea | 0-12 years | Microscopic examination | Iraq | Asia | 500 | 3 | 9 | [180] |
| Ashour et al. | 2021 | Children with diarrhea | 1-12 years | Microscopic examination | Iraq | Asia | 926 | 52 | 9 | [181] |
| Hu et al. | 2022 | Children | 0-12years | Microscopic examination | China | Asia | 11575 | 11 | 10 | [182] |

NR: not report

QA: quality assessment

**References**

1. Khansari AMa: **Investigation of intestinal parasite infection in elementary school students of Absard region in November 1996**. *Research in Medicine* 2000, **24**(3):177-190.

2. Youssef M, Shurman A, Bougnoux M-E, Rawashdeh M, Bretagne S, Strockbine N: **Bacterial, viral and parasitic enteric pathogens associated with acute diarrhea in hospitalized children from northern Jordan**. *FEMS Immunology & Medical Microbiology* 2000, **28**(3):257-263.

3. Kalantari N, Mobadi I: **Enteral parasitic contamination in Babol day-care center, 1997**. *Journal of Babol University Of Medical Sciences* 2000, **2**(5):57-60.

4. al. Re: **Parasitic infections in school children of Ray city**. *Journal of Faculty of Medicine, Tehran University of Medical Sciences* 2000, **58**(1):82-86.

5. Shahabi S: **Epidemiologic Survey of Intestinal Parasites in School Children of Shahriar in 1993**. *Research in Medicine* 2000, **24**(2):133-139.

6. Arif SM, Ibrahim ZA: **Survey on the prevalence of intestinal parasites among orphan children inhabit two state homes in Baghdad city**. *Bulletin of the Iraq Natural History Museum* 2001, **9**(3).

7. Saifi M: **Wajihullah. Intestinal parasitic infestation in school children of Ujhani, Budaun**. *J Parasit Dis* 2001, **25**:26-29.

8. SAIDI JM, Sajadi S: **Study of the parasitic infections of school children in rural areas of Hamadan**. 2001.

9. Ghahramanloo M, Hassanjani Roshan M, Haji Ahmadi M: **Prevalence of intestinal parasites in primary school children, Eastern Bandpay, Babol, 1999**. *Journal of Babol University of Medical Sciences* 2001, **3**(2):47-51.

10. Mahdi NK, Ali NH: **Intestinal parasitic (Including Cryptosporidium) infections in day-care centres**. *Bahrain Medical Bulletin* 2002, **24**(4):135-137.

11. Kaur R, Rawat D, Kakkar M, Uppal B, Sharma V: **Intestinal parasites in children with diarrhea in Delhi, India**. *Southeast Asian journal of tropical medicine and public health* 2002, **33**(4):725-729.

12. Lee K-J, Bae Y-T, Kim D-H, Deung Y-K, Ryang Y-S, Kim H-J, Im K-I, Yong T-S: **Status of intestinal parasites infection among primary school children in Kampongcham, Cambodia**. *The Korean journal of parasitology* 2002, **40**(3):153-155.

13. Moghimi M, Sharifi A: **Prevalence of Intestinal Parasites in Preschool Children of Yasuj (1380-1381)**. 2002.

14. Fernandez MC, Verghese S, Bhuvaneswari R, Elizabeth S, Mathew T, Anitha A, Chitra A: **A comparative study of the intestinal parasites prevalent among children living in rural and urban settings in and around Chennai**. *The Journal of communicable diseases* 2002, **34**(1):35-39.

15. Davami MH, Khazaii MR, Eslamirad Z, Mostofi M, Modaresi M: **An investigation on intestinal parasitic infections and the effective demographic factors in children (1-3 years) in Shahrak-e-Valiasr Arak during 1999**. *Journal of Arak University of Medical Sciences* 2002, **5**(2):5-10.

16. Al-Hindi AI: **Prevalence of intestinal parasites among school children in Deir El-Balah Town in Gaza Strip, Palestine**. *Annals of Saudi Medicine* 2002, **22**(3-4):273-275.

17. Waikagul J, Krudsood S, Radomyos P, Radomyos B, Chalemrut K, Jonsuksuntigul P, Kojima S, Looareesuwan S, Thaineau W: **A cross-sectional study of intestinal parasitic infections among schoolchildren in Nan Province, Northern Thailand**. *Southeast Asian Journal of Tropical Medicine and Public Health* 2002, **33**(2):218-223.

18. SN W, MA B: **Intestinal parasitic infections in children from Northern Pakistan**. 2003.

19. Widajanti E, Garna H, Chairulfatah A, Hudaya D: **Giardiasis in daycare centers at Rancabali tea plantation, Bandung District, Indonesia**. *Paediatrica Indonesiana* 2003, **43**(5):158-161.

20. Saksirisampant W, Nuchprayoon S, Wiwanitkit V, Yenthakam S, Ampavasiri A: **Intestinal parasitic infestations among children in an orphanage in Pathum Thani province**. *Journal of the Medical Association of Thailand* 2003, **86**(Suppl 2):S263-S270.

21. Hosain GM, Saha S, Begum A: **Impact of sanitation and health education on intestinal parasite infection among primary school aged children of Sherpur, Bangladesh**. *Tropical doctor* 2003, **33**(3):139-143.

22. AHMAD RR, Varzandeh F, Arab M, Abbaszadeh A: **Prevalence of intestinal parasite infections in the day care centers of bam**. 2003.

23. Piangjai S, Sukontason K, Sukontason KL: **Intestinal parasitic infections in hill-tribe schoolchildren in Chiang Mai, northern Thailand**. *Southeast Asian journal of tropical medicine and public health* 2003, **34**:90-93.

24. Heidari A, Rokni M: **Prevalence of intestinal parasites among children in day-care centers in Damghan-Iran**. *Iranian Journal of Public Health* 2003, **32**(1):31-34.

25. Azazy A, Raja'a Y: **Malaria and intestinal parasitosis among children presenting to the paediatric centre in Sana'a, Yemen**. *EMHJ-Eastern Mediterranean Health Journal, 9 (5-6), 1048-1053, 2003* 2003.

26. Chandrasena T, de Alwis A, De Silva L, Morel R, De Silva N: **Intestinal parasitoses and the nutritional status of Veddah children in Sri Lanka**. *Southeast Asian journal of tropical medicine and public health* 2004, **35**:255-259.

27. Baldo ET, Belizario VY, De Leon WU, Kong H-H, Chung D-I: **Infection status of intestinal parasites in children living in residential institutions in Metro Manila, the Philippines**. *The Korean Journal of Parasitology* 2004, **42**(2):67.

28. Zakai H: **Intestinal parasitic infections among primary school children in Jeddah, Saudi Arabia**. *Journal of the Egyptian Society of Parasitology* 2004, **34**(3):783-790.

29. Chaudhry ZH, Afzal M, Malik MA: **Epidemiological factors affecting prevalence of intestinal parasites in children of Muzaffarabad district**. *Pakistan Journal of Zoology* 2004, **36**(4):267-271.

30. Rai K, Sherchand JB, Bhatta DR, Bhattarai NR: **Status of Giardia intestinalis infection among the children attending Kanti children hospital, Nepal**. *Scientific World* 2005, **3**(3):102-105.

31. Taheri F, FESHAREKINIA A, SAADATJOO S: **STUDY OF PREVALENCE OF INTESTINAL PARASITIC INFECTION AMONG 6 YEAR OLD CHILDREN IN BIRJAND**. 2004.

32. Sharma BK, Rai SK, Rai DR, Choudhury DR: **Prevalence of intestinal parasitic infestation in school children in the northeastern part of Kathmandu Valley, Nepal**. *Age* 2004, **4**(10):11-14.

33. Saksirisampant W, Prownebon J, Kanmarnee P, Thaisom S, Yenthakam S, Nuchprayoon S: **Prevalence of parasitism among students of the Karen hill-tribe in Mae Chame district, Chiang Mai province, Thailand**. *Journal of the Medical Association of Thailand= Chotmaihet Thangphaet* 2004, **87**:S278-283.

34. Park SK, Kim D-H, Deung Y-K, Kim H-J, Yang E-J, Lim S-J, Ryang Y-S, Jin D, Lee K-J: **Status of intestinal parasite infections among children in Bat Dambang, Cambodia**. *The Korean Journal of Parasitology* 2004, **42**(4):201-203.

35. Astal Z: **Epidemiological survey of the prevalence of parasites among children in Khan Younis governorate, Palestine**. *Parasitology research* 2004, **94**(6):449-451.

36. Adnan Mohamad A, Samih Sami A: **Intestinal parasitic infection among primary school children in south Jordan**. 2004.

37. Ashtiani M, MAHJOUB F, Kashi L: **GIARDIASIS AND OTHER PARASITIC INFECTIONS IN STOOL SPECIMENS, DUODENAL BIOPSY AND DUODENAL ASPIRATION IN CHILDREN**. 2004.

38. Chandrasena T, Hapuarachchi H, Dayanath M, de Silva N: **Intestinal parasitoses and the nutritional status of internally displaced children in Vavuniya**. 2005.

39. Ahsan-ul-Wadood BA, Rhman A, Qasim K: **Frequency of intestinal parasite infestation in Children Hospital Quetta**. *Pakistan Journal of Medical Research* 2005, **44**(2):87-88.

40. Sadjjadi S, Tanideh N: **Nutritional Status of Preschool Children Infected with Giardia intestinalis**. *Iranian J Publ Health* 2005, **34**(4):51-57.

41. Basima A: **Study on the Prevalence of Intestinal Parasites Among Children Attending Al-Daura Health Centre-Baghdad**. *Iraqi National Journal of Nursing Specialties* 2005, **18**(1).

42. Daryani A, Ettehad GH: **Prevalence of Intestinal Infestation among Primary School Students in Ardabil, 2003**. *Journal of Ardabil University of Medical Sciences* 2005, **5**(3):229-234.

43. Easow JM, Mukhopadhyay C, Wilson G, Guha S, Jalan BY, Shivananda PG: **Emerging opportunistic protozoa and intestinal pathogenic protozoal infestation profile in children of western Nepal**. *Nepal Medical College journal: NMCJ* 2005, **7**(2):134-137.

44. Mosaviani Z: **Contamination with Oxyure and Giardia in children of kindergartens and welfare centers in Tehran**. *Avicenna Journal of Nursing and Midwifery Care* 2006, **14**(1):40-50.

45. al. ANe: **Investigating the prevalence of intestinal parasitic infections and related factors in kindergarten and primary school children in the urban areas of Semnan province (2014)**. *Scientific journal of Semnan University of Medical Sciences* 2006, **1**.

46. Chhakda T, Muth S, Socheat D, Odermatt P: **Intestinal parasites in school-aged children in villages bordering Tonle Sap Lake, Cambodia**. *Southeast Asian journal of tropical medicine and public health* 2006, **37**(5):859.

47. Saksirisampant W, Prownebon J, Kulkumthorn M, Yenthakam S, Janpla S, Nuchprayoon S: **Prevalence of intestinal parasitic infections among school children in the central region of Thailand**. *Journal of the Medical Association of Thailand= Chotmaihet Thangphaet* 2006, **89**(11):1928-1933.

48. Al Saeed A, Issa S: **Frequency of Giardia lamblia among children in Dohuk, northern Iraq**. *EMHJ-Eastern Mediterranean Health Journal, 12 (5), 555-561, 2006* 2006.

49. kh Al Jarousha AM: **Incidence of Intestinal Parasites among the School Children of Gaza Strip**. 2006.

50. Das R, Kumar PS, Biswas R: **Prevalence of intestinal-parasites and its association with sociodemographic, environmental and behavioral factors in children in Pokhara valley, Nepal**. *African Journal of Clinical and Experimental Microbiology* 2006, **7**(2):106-115.

51. F Mohseni M, B Shahidi Z: **Prevalence of giardiasis in daycare children at rafsanjan nurseries in 2003**. 2007.

52. Aminzadeh Z, Tarami M, Gachkar L: **Prevalence of intestinal parasites and related factors in primary school children in Varamin**. *Journal of Comprehensive Pediatrics* 2007, **1**(2):55-58.

53. Wongstitwilairoong B, Srijan A, Serichantalergs O, Fukuda CD, McDaniel P, Bodhidatta L, Mason CJ: **Intestinal parasitic infections among pre-school children in Sangkhlaburi, Thailand**. *The American journal of tropical medicine and hygiene* 2007, **76**(2):345-350.

54. Ebadi M, Behravan F, Moghaddam SH: **Prevalence of intestinal parasites and clinical manifestations in children**. *Iranian Journal of Public Health* 2007, **36**(Supple 2):1-2.

55. Ngrenngarmlert W, Lamom C, Pasuralertsakul S, Yaicharoen R, Wongjindanon N, Sripochang S, Suwajeejarun T, Sermsart B-O, Kiatfuengfoo R: **Intestinal parasitic infections among school children in Thailand**. *Trop Biomed* 2007, **24**(2):83-88.

56. Mehraj V, Hatcher J, Akhtar S, Rafique G, Beg MA: **Prevalence and factors associated with intestinal parasitic infection among children in an urban slum of Karachi**. *PloS one* 2008, **3**(11):e3680.

57. Younas M, Shah S, Talaat A: **Frequency of Giardia lamblia infection in children with recurrent abdominal pain**. *Journal-Pakistan Medical Association* 2008, **58**(4):171.

58. AL-Rubaee RJ: **Prevalence of parasitic infestation among primary school children in Thi-Qar governorate**. *University of Thi-Qar Journal Of Medicine* 2008, **2**(1):63-68.

59. Davami MH, Roohi R, Sadeghi AR: **The Prevalence of intestinal parasitic infections among 7-15 year old children in Jahrom, Iran during 2006-7**. *Journal of Jahrom University of Medical Sciences* 2008, **6**(1):49-55.

60. Al-Braiken FA: **Is intestinal parasitic infection still a public health concern among Saudi children?** *Saudi medical journal* 2008, **29**(11):1630-1635.

61. FALAHI S, GORAVI M, GHARAHGOUZLOU B, Sepahvand A, Mahouti F: **A comparative evaluation of giardiasis prevalence by rutine parasitical assays and antigen detection in elementary school children in Delfan town, Iran**. 2008.

62. Mahfoth N, S Al-Zako S, I Ftohe Z: **Study on Intestinal Parasites as a Causative of Diarrhea and Some Effectors on Them in Children of Neinavah Governorate**. *Rafidain Journal of Science* 2008, **19**(4):37-50.

63. Al Hindi A, El Kichaoi A: **Occurrence of gastrointestinal parasites among pre-school children, Gaza, Palestine. The Islamic University Journal, Vol. 16, No. 1**. In*.*: ISSN; 2008.

64. Almerie MQ, Azzouz MS, Abdessamad MA, Mouchli MA, Sakbani MW, Alsibai MS, Alkafri A, Ismail MT: **Prevalence and risk factors for giardiasis among primary school children in Damascus, Syria**. *Saudi medical journal* 2008, **29**(2):234.

65. Mohammed NA: **Prevalence of Intestinal Parasites among Children (1-13) Years Age in Baquba City**. *Al-Mustansiriyah Journal of Science* 2008, **19**(2).

66. Yusof H, Ghani MKA: **Giardiasis Among Orang Asli Children at Pos Lenjang, Pahang**. *Malaysian Journal of Health Sciences* 2009:97-102.

67. Mumtaz S, Siddiqui H, Ashfaq T: **Frequency and risk factors for intestinal parasitic infection in children under five years age at a tertiary care hospital in Karachi**. *J Pak Med Assoc* 2009, **87**:32.

68. Shrestha SK, Rai SK, Vitrakoti R, Pokharel P: **Parasitic infection in school children in Thimi area, Kathmandu valley**. *J Nepal Assoc Med Lab Sci* 2009, **10**(1):31-33.

69. Ajjampur SS, Sankaran P, Kannan A, Sathyakumar K, Sarkar R, Gladstone BP, Kang G: **Giardia duodenalis assemblages associated with diarrhea in children in South India identified by PCR-RFLP**. *The American journal of tropical medicine and hygiene* 2009, **80**(1):16.

70. Hassen TF: **Spread of Giardiasis Among Children In Al-Nassiria city southern Iraq**. *JOURNAL OF THI-QAR SCIENCE* 2009, **1**(3).

71. Jasim TM, Al-Mugdadi SFH: **The incidence of Entamoeba histolytica & Giardia Lamblia associated with diarrhea among children in lbn Al-Balady Hospital in Baghdad**. *infection* 2009, **7**:8.

72. Gyawali N, Amatya R, Nepal H: **Intestinal parasitosis in school going children of Dharan municipality, Nepal**. *Tropical Gastroenterology* 2009, **30**:145-147.

73. Rayan P, Verghese S, McDonnell PA: **Geographical location and age affects the incidence of parasitic infestations in school children**. *Indian Journal of Pathology and Microbiology* 2010, **53**(3):498.

74. Khan IA: **A CLINICO-EPIDEMIOLOGICAL STUDY OF GIARDIASIS IN CHILDREN IN RURAL PUNJAB, PAKISTAN**.

75. Hasan SF: **Intestinal Parasites in Children under Five Years with Diarrhea in Kerbala, Iraq**. *Journal of Kerbala University* 2010, **8**(1):415-420.

76. khudair Hussein T: **Prevalence and related risk factors for Giardia lamblia infection among children with acute diarrhea in thi-qar, southern Iraq**. *Thi-Qar Medical Journal (TQMJ)* 2010, **4**(4):201068-201074.

77. Al-Haddad A, Baswaid S: **Frequency of intestinal parasitic infection among children in Hadhramout governorate (Yemen)**. *J Egypt Soc Parasitol* 2010, **40**(2):479-488.

78. Mohammad Taher I, Mohannad M, Susan AD, Nahla AH, Raghad S, Bishr D, Mohammad AM: **Prevalence of intestinal parasites in children of elementary educational level in rural of daraa Governorate-Syria**. 2010.

79. Al-Mohammed HI: **Genotypes of Giardia intestinalis clinical isolates of gastrointestinal symptomatic and asymptomatic Saudi children**. *Parasitology research* 2011, **108**(6):1375-1381.

80. Ajjampur S, Koshy B, Venkataramani M, Sarkar R, Joseph A, Jacob K, Ward H, Kang G: **Effect of cryptosporidial and giardial diarrhoea on social maturity, intelligence and physical growth in children in a semi-urban slum in south India**. *Annals of tropical paediatrics* 2011, **31**(3):205-212.

81. Boontanom P, Mungthin M, Tan-Ariya P, Naaglor T, Leelayoova S: **Epidemiology of giardiasis and genotypic characterization of Giardia duodenalis in preschool children of a rural community, central Thailand**. *Trop Biomed* 2011, **28**(1):32-39.

82. Suman M, Alam M, Pun S, Khair A, Ahmed S, Uchida R: **Prevalence of Giardia lamblia infection in children and calves in Bangladesh**. *Bangladesh Journal of Veterinary Medicine* 2011, **9**(2):177-182.

83. Rashid M, Joshi M, Joshi H, Fatemi K: **Prevalence of Intestinal Parasites among School Going Children In Bareilly District**. *National Journal of Integrated Research in Medicine* 2011, **2**(1).

84. Bisht D, Verma AK, Bharadwaj HHD: **Intestinal parasitic infestation among children in a semi-urban Indian population**. *Tropical parasitology* 2011, **1**(2):104.

85. Kadir MA, Mohammad-Ali SM: **Nutritional status of children infected with Giardia lamblia and Entamoeba histolytica infections in Kalar town, Iraq**. *Tikri J Pharm Scie* 2011, **7**(2):162-170.

86. flah Nazal M, Jawad SJ, Khudair MK: **Study the Causes of Parasitic Diarrhea in Children in Al-Ahded Village. Dyala governarate**. *Baghdad Science Journal* 2011, **8**(4).

87. Jaeffer HS: **Prevalence of Gairdia lamblia and Entamoeba histolytic/Entamoeba dispare infections among children in AL-Shulaa and AL-Khadimya-Baghdad-Iraq**. *J Univ Anbar Pure Sci* 2011, **5**:6-10.

88. Matthys B, Bobieva M, Karimova G, Mengliboeva Z, Jean-Richard V, Hoimnazarova M, Kurbonova M, Lohourignon LK, Utzinger J, Wyss K: **Prevalence and risk factors of helminths and intestinal protozoa infections among children from primary schools in western Tajikistan**. *Parasites & vectors* 2011, **4**(1):1-13.

89. Aher A, Kulkarni S: **Prevalence of intestinal parasites in school going children in a rural community**. *Int J Biomed Res* 2011, **2**(12):605-607.

90. MT I: **< The> species of intestinal parasites and their prevalence in children in elementary educational level in rural of Damascus-Kalamoon-Syria**. 2011.

91. Hussein AS: **Prevalence of intestinal parasites among school children in northern districts of West Bank‐Palestine**. *Tropical Medicine & International Health* 2011, **16**(2):240-244.

92. Abbas NF, El-Shaikh KA, Almohammady MS: **Prevalence of Giardia lamblia in diarrheic children in Almadinah Almunawarh, KSA**. *Journal of Taibah University for Science* 2011, **5**:25-30.

93. Taheri F, Namakin K, Zarban A, Sharifzadeh G: **Intestinal parasitic infection among school children in South Khorasan Province, Iran**. 2011.

94. Ashtiani M, Monajemzadeh M, Saghi B, Shams S, Mortazavi S, Khaki S, Mohseni N, Kashi L, Nikmanesh B: **Prevalence of intestinal parasites among children referred to Children’s Medical Center during 18 years (1991–2008), Tehran, Iran**. *Annals of Tropical Medicine & Parasitology* 2011, **105**(7):507-513.

95. Khan MS, Jehan S, Akram M, Rabnwaz MZ, Lathif Z, Hussain F, Naeem M: **Prevalence of intestinal protozoan and worm infestation in primary school going children of 5-10 years of age in district Bannu**. *Annals of Pakistan Institute of Medical Sciences* 2012, **8**:243-248.

96. Hammadi: **study for intestinal parasites among children in AL-mahmoudyia area/Baghdad province**. *Al-Kufa University Journal for Biology* 2012, **4**(1).

97. Baig MF, Kharal SA, Qadeer SA, Badvi JA: **A comparative study of different methods used in the detection of Giardia lamblia on fecal specimens of children**. *Annals of Tropical Medicine & Public Health* 2012, **5**(3).

98. Shrestha A, Narayan K, Sharma R: **Prevalence of intestinal parasitosis among school children in Baglung District of Western Nepal**. *Kathmandu university medical journal* 2012, **10**(1):62-65.

99. Saksirisampant W, Boontanom P, Mungthin M, Tan-Ariya P, Lamchuan D, Siripattanapipong S, Leelayoova S: **Prevalence of giardiasis and genotypic characterization of Giardia duodenalis in hilltribe children, Northern Thailand**. *Trop Biomed* 2012, **29**(3):331-338.

100. Rostami M, Tohidi F, Sharbatkhori M, Taherkhani H, Eteraf A, Mohammadi R, Maghsoodloorad F: **The prevalence of intestinal parasitic infections in primary school students in Gorgan, Iran**. *Medical Laboratory Journal* 2012, **6**(2):42-46.

101. Tiwary SK, Singh BK, Sharan S, Sinha SK, Kumar R: **Prevalence of Intestinal Parasitic Infection in Children of Slum Area of Dhanbad, Jharkhand**.

102. Qays Ibrahim A: **Prevalence of Entamoeba histolytica and Giardia lamblia in Children in Kadhmiyah Hospital**. *The Iraqi Journal of Veterinary Medicine (IJVM)* 2012, **36**(1):32-36.

103. Moore CE, Hor PC, Soeng S, Sun S, Lee SJ, Parry CM, Day NP, Stoesser N: **Changing patterns of gastrointestinal parasite infections in Cambodian children: 2006–2011**. *Journal of tropical pediatrics* 2012, **58**(6):509-512.

104. Khavari DH, Rahimkhani M: **Intestinal infections in malnourished children in south of Tehran, Iran**. *African Journal of Microbiology Research* 2013, **7**(22):2741-2744.

105. Zabolinejad N, Berenji F, Bayati Eshkaftaki E, Badeii Z, Banihashem A, Afzalaqaei M: **Intestinal parasites in children with lymphohematopoietic malignancy in Iran, Mashhad**. *Jundishapur Journal of Microbiology* 2013, **6**(6).

106. Khadka KS, Kaphle HP, Gurung K, Shah Y, Sigdel M: **Study of intestinal parasitosis among school going children in Pokhara, Nepal**. *Journal of Health and Allied Sciences* 2013, **3**(1):47-50.

107. Maharjan R, Timilshina M, Shakya R, Bhattarai S, Gurung P: **Prevalence of intestinal parasitic infection of kindergarten children**. *International Journal of Infection and Microbiology* 2013, **2**(3):111-113.

108. Prownebon J, Charupoonphol P, Saksirisampant P, Limvorapitak T, Seepongpun U, Saksirisampant W: **Intestinal parasitic infections: high prevalence of Giardia intestinalis in children living in an orphanage compared with hill-tribe children as detected by microscopy and ELISA**. *Asian Biomedicine* 2013, **7**(6):855-863.

109. Pradhan P, Bhandary S, Shakya P, Acharya T, Shrestha A: **Prevalence of intestinal parasitic infections among public school children in a rural village of Kathmandu Valley**. *Nepal Med Coll J* 2014, **16**(1):50-53.

110. Sah RB, Bhattarai S, Yadav S, Baral R, Jha N, Pokharel PK: **A study of prevalence of intestinal parasites and associated risk factors among the school children of Itahari, Eastern Region of Nepal**. *Tropical parasitology* 2013, **3**(2):140.

111. Ashok R, Suguneswari G, Satish K, Kesavaram V: **Prevalence of intestinal parasitic infection in school going children in Amalapuram, Andhra Pradesh, India**. *Shiraz E-Medical Journal* 2013, **14**(4).

112. Patel P, Chaudhary U, CHUDASAMA R: **Intestinal parasites prevalence and related factors in hospitalized children age upto 12 years with diarrhea in Surat, India**. *Journal of Pediatric Sciences* 2013, **5**.

113. Salman YJ, Mussttafa MI: **Evaluation of the Employment of Four Laboratory Diagnostic Methods in Detecting of Giardia lamblia among Children in Kirkuk City**. *Journal of Kirkuk Medical College* 2021, **1**(2):52-60.

114. Al Ayed MS, Asaad AM, Mahdi AA, Qureshi MA: **Aetiology of acute gastroenteritis in children in Najran region, Saudi Arabia**. *J Health Specialties* 2013, **1**:84.

115. Al-Mekhlafi HM, Al-Maktari MT, Jani R, Ahmed A, Anuar TS, Moktar N, Mahdy MA, Lim YA, Mahmud R, Surin J: **Burden of Giardia duodenalis infection and its adverse effects on growth of schoolchildren in rural Malaysia**. *PLoS neglected tropical diseases* 2013, **7**(10):e2516.

116. Momen Heravi M, Rasti S, Vakili Z, Moraveji A, Hosseini F: **Prevalence of intestinal parasites infections among Afghan children of primary and junior high schools residing Kashan city, Iran, 2009-2010**. *Iranian Journal of Medical Microbiology* 2013, **7**(1):46-52.

117. Tiwari BR, Chaudhary R, Adhikari N, Jayaswal SK, Poudel TP, Rijal KR: **Prevalence of intestinal parasitic infections among school children of Dadeldhura District, Nepal**. *Journal of Health and Allied Sciences* 2013, **3**(1):14-16.

118. Zangana NKA: **Distribution of intestinal protozoa and the effect of Giardia lamblia on anthropometric measurement in school children in kirkuk**. *Tikrit Medical Journal* 2013, **19**(1):55-68.

119. Mero W, Hussein JN: **Prevalence of Intestinal Parasites Among Children in Various Localities of Duhok City and Nearby Villages**. *Science Journal of University of Zakho* 2013, **1**(1):189-199.

120. Tandukar S, Ansari S, Adhikari N, Shrestha A, Gautam J, Sharma B, Rajbhandari D, Gautam S, Nepal HP, Sherchand JB: **Intestinal parasitosis in school children of Lalitpur district of Nepal**. *BMC research notes* 2013, **6**(1):1-6.

121. PISHKARIE AR, AGDAM SM: **GIARDIASIS AND OTHER PARASITIC INFECTIONSIN STOOL SPECIMENS, IN CHILDREN**. 2013.

122. Kazemian H, Shavalipour A, Mohebi R, Ghafurian S, Aslani S, Maleki A, Kardan J, Heidari H, Sadeghifard N: **Estimation of the parasitic infection prevalence in children with Helicobacter pylori infection in Ilam city (2012-2013)**. *Archives of Pediatric Infectious Diseases* 2014, **2**(3).

123. Torabi Z, Niksirat A, Mazloomzadeh S, Ahmadiafshar A: **Consistency of direct microscopic examination and ELISA in detection of Giardia in stool specimen among children**. *Asian Pacific Journal of Tropical Disease* 2014, **4**:S725-S727.

124. Muhsen K, Cohen D, Levine MM: **Can Giardia lamblia infection lower the risk of acute diarrhea among preschool children?** *Journal of tropical pediatrics* 2014, **60**(2):99-103.

125. Saeed A, Khairi NM: **Study The Prevalence Of Giardiasis and Cryptosporidiosis Among Children At Al-Ressafa Side of Baghdad By Comparison Between The Efficiency Of Some Diagnostic Methods**. *AL-Taqani* 2014, **27**(2):82-93.

126. Anvari TMH, Mirjalili MM, Aghabagheri M: **Prevalence of intestinal parasites in children attending day-care centers in yazd city, iran**. 2014.

127. Ullah W, Shah A, Jamal Q, Ullah S, Muhammad I, Ullah H: **Prevalence of intestinal parasites among school children in District Upper Dir, Khyber Pakhtunkhwa Pakistan**. *IJB* 2014, **5**(1):1-8.

128. Mane M, Kadu A, Mumbre S, Deshpande M, Gangurde N: **Prevalence of intestinal parasitic infections and associated risk factors among pre-school children in tribal villages of North Maharashtra, India**. *Int J Res Health Sci* 2014, **2**(1):133-139.

129. Al-Delaimy AK, Al-Mekhlafi HM, Nasr NA, Sady H, Atroosh WM, Nashiry M, Anuar TS, Moktar N, Lim YA, Mahmud R: **Epidemiology of intestinal polyparasitism among Orang Asli school children in rural Malaysia**. *PLoS neglected tropical diseases* 2014, **8**(8):e3074.

130. Ahmed T, Khanum H, Barua P, Arju T, Uddin MS, Haque R: **Detection of Giardia lamblia in children by microscopy, ELISA and real time PCR assay**. *Dhaka University Journal of Biological Sciences* 2014, **23**(2):197-204.

131. Kiran T, Shashwati N, Vishal B, Kumar DA: **Intestinal Parasitic infections and Demographic status of school children in Bhopal region of Central India**. *IOSR J Pharm Biol Sci* 2014, **9**:83-87.

132. Abdi J, Farhadi M, Aghaee S: **Prevalence of intestinal parasites among children attending the daycare centers of Ilam, western Iran**. *Journal of Medical Sciences (Faisalabad)* 2014, **14**(3):143-146.

133. Tappeh KH, Manafi G, Asgharzadeh M, Manafi F: **Incidence of Giardia lamblia subspecies by PCR-RFLP in stool specimens of hospitalized children at Urmia Mutahhari hospital, West Azerbaijan province, Iran**. *Iranian Journal of Parasitology* 2014, **9**(4):541.

134. Haji Aliani F, Einipor S, Abadi A, Tahvildar Bidrouni F: **Consideration of intestinal parasite in day-care center children in Karaj city in 2012**. *Alborz University Medical Journal* 2014, **3**(4):239-252.

135. Hamza HM, Al-Ibrahimi LA-K: **Molecular Diagnosis of Giardia intestinal parasite for children with diarrhea by using Real-Time PCR technique**. *Al-Qadisiyah Journal of Pure Science* 2014, **19**(4):29-41.

136. Waqar A, Hassanain A, Alyaa A-k: **Intestinal parasitic diarrhea among children in Baghdad–Iraq**. *Tropical biomedicine* 2014, **31**(3):499-506.

137. Ben-Shimol S, Sagi O, Greenberg D: **Differences in prevalence of parasites in stool samples between three distinct ethnic pediatric populations in southern Israel, 2007–2011**. *Parasitology international* 2014, **63**(2):456-462.

138. Singh S: **Study of the Prevalence of Intestinal Parasitic Infection in Children of Ghaziabad**. *Microbiology* 2015, **4**(2).

139. Hussein RA, Al-Mayah QS, Merdaw MA-z, Al-Bashier NT, Abd Al-Abbas A, Jasem IA: **Evaluation of multiplex real-time PCR and ELISA in detection of intestinal protozoan parasites from children with diarrheal disease**. *International Journal* 2015, **3**(9):782-788.

140. Pandey S, Lo AL, Shrestha RB: **Intestinal parasitic infections among school children of Northern Kathmandu, Nepal**. *Asian Pacific Journal of Tropical Disease* 2015, **5**:S89-S92.

141. Sedighi I, Asadi M, Olfat M, Maghsood AH: **Prevalence and risk factors of Giardia lamblia and Blastocystis hominis infections in children under ten years old, Hamadan, Iran**. *Avicenna Journal of Clinical Microbiology and Infection* 2015, **2**(2):22713-22713.

142. Faraji R, Ahmadian F, Javadi GR, Barshahi PM: **Prevalence of Giardiasis among children in childcare centers in Kermanshah, Iran**. *Int J Res Med Sci* 2015, **3**(7):1717-1720.

143. Moore CE, Nget P, Saroeun M, Kuong S, Chanthou S, Kumar V, Bousfield R, Nader J, Bailey JW, Beeching NJ: **Intestinal parasite infections in symptomatic children attending hospital in Siem Reap, Cambodia**. *PloS one* 2015, **10**(5):e0123719.

144. gharavi A: **Study of Prevalence of intestinal parasites infection among children in & attending to Karbala teaching hospital for children**. *journal of kerbala university* 2015, **13**(1).

145. Erzaiq ZS: **The Frequency of Giardia lamblia Infection among Children with Malnutrition at General Pediatrics Hospital in Kirkuk City**. *Tikrit Medical Journal* 2016, **21**(1).

146. Ying-fang Y, Xiu-ping W, Yan-hong C, Jia-xu C, Li-guang T: **Infection of Giardia lamblia in HIV-Infected Individuals and in Kindergarden Children in Rural Area of Anhui and Genotype Analysis**. *CHINESE JOURNAL OF PARASITOLOGY AND PARASITIC DISEASES* 2016, **34**(6):11.

147. Omer S, Alanazi A: **Coprologic and molecular prevalence of Giardia duodenalis from children in Tabuk City, Saudi Arabia**. *Tropical Biomedicine* 2016, **33**(2):320-326.

148. Alsubaie ASR, Azazy AA, Omer EO, Al-Shibani LA, Al-Mekhlafi AQ, Al-Khawlani FA: **Pattern of parasitic infections as public health problem among school children: A comparative study between rural and urban areas**. *Journal of Taibah University Medical Sciences* 2016, **11**(1):13-18.

149. Ahmed T, Khanum H, Uddin MS, Barua P, Arju T, Kabir M, Haque R: **Entamoeba histolytica, Giardia lamblia and Cryptosporidium spp. infection in children in an urban slum area of Bangladesh**. *Bioresearch Communications-(BRC)* 2016, **2**(1):175-181.

150. Moore CE, Elwin K, Seng C, Mao S, Suy K, Kumar V, Nader J, Bousfield R, Perera S, Bailey JW: **Molecular characterization of Cryptosporidium species and Giardia duodenalis from symptomatic Cambodian children**. *PLoS neglected tropical diseases* 2016, **10**(7):e0004822.

151. Kiani H, Haghighi A, Salehi R, Azargashb E: **Distribution and risk factors associated with intestinal parasite infections among children with gastrointestinal disorders**. *Gastroenterology and hepatology from bed to bench* 2016, **9**(Suppl1):S80.

152. M Abdul-Hussein S: **Cryptosporidium and Giardia infection in children with malignancies in Basrah**. *The Medical Journal of Basrah University* 2017, **35**(1):17-26.

153. Babakhani M, Safari R, Rajati F, Salimi S: **Prevalence and risk factors associated with intestinal parasitic infections among school children in Gashky, West of Iran**. *International Journal of Pediatrics*, **5**(7):5263-5273.

154. Lass A, Karanis P, Korzeniewski K: **First detection and genotyping of Giardia intestinalis in stool samples collected from children in Ghazni Province, eastern Afghanistan and evaluation of the PCR assay in formalin-fixed specimens**. *Parasitology research* 2017, **116**(8):2255-2264.

155. Wang T, Fan Y, Koehler AV, Ma G, Li T, Hu M, Gasser RB: **First survey of Cryptosporidium, Giardia and Enterocytozoon in diarrhoeic children from Wuhan, China**. *Infection, Genetics and Evolution* 2017, **51**:127-131.

156. Effati F, Dalimi A, Pirestani M: **A survey on Giardia and Cryptosporidium infection and genotyping common Giardia in children in Alborz Province**. *Pathobiology Research* 2018, **21**(3):133-139.

157. Al-Abodi HRJ: **Effect and spread of Giardia parasite on children in primary development stages in southern Iraq**. *Biochem Cell Arch* 2018, **18**(2):1537-1541.

158. Qayyum H, Qayyum F, Naeem H: **Frequency Of Giardiasis In 5 to 12 years old Children With Recurrent Abdominal Pain**. *Rawal Medical Journal* 2018, **43**(4):670-670.

159. Babat S, Sirekbasan S, Macin S, Kariptas E, Polat E: **Diagnostics of intestinal parasites by light microscopy among the population of children between the ages of 4-12 in eastern Turkey**. 2018.

160. Chanu N, Singh T, Dutta S: **Detection and genetic characterization of Giardia intestinalis in children with gastrointestinal symptoms by PCR RFLP in Sikkim, India**. *Journal of Natural Science, Biology and Medicine* 2018, **9**(2).

161. Kasaei R, Carmena D, Jelowdar A, Beiromvand M: **Molecular genotyping of Giardia duodenalis in children from Behbahan, southwestern Iran**. *Parasitology research* 2018, **117**(5):1425-1431.

162. Singh BK, Sharan S, Jaiswal NK, Kumar R: **A Study on the Prevalence of Giardia lamblia Infection in Children among the Population of Dhanbad, A Coal Field Area**. *Int J Curr Microbiol App Sci* 2018, **7**(7):3552-3555.

163. Bakr M, Mohammad SA, Kadir M: **Distribution of Giardia lamblia Among local and Displaced Children in Kirkuk City**. *Tikrit Journal of Pure Science* 2018, **23**(9):28-31.

164. Hafeez S, Ali Z, Zafar A: **Prevalence of Intestinal Parasitic Infestation at Children Hospital of Lahore**. *Pak J Med Health scien* 2018, **12**(2):645-648.

165. Naz A, Nawaz Z, Rasool MH, Zahoor MA: **Cross-sectional epidemiological investigations of Giardia lamblia in children in Pakistan**. *Sao Paulo Medical Journal* 2018, **136**:449-453.

166. Kadir MA, El-Yassin ST, Ali A: **Detection of Entamoeba histolytica and Giardia lamblia in children with diarrhea in Tikrit city**. *Tikrit Journal of Pure Science* 2018, **23**(6):57-64.

167. Rather SH, Rather AH: **Prevalence of parasitic infestation among school going children of South-Western Kashmir valley**. 2019.

168. Jeyaprakasam NK, Abd Ghani MK: **Giardiasis amongst the Aboriginal School Children at Sungai Raba Village Gerik, Perak, Malaysia**. *International Medical Journal* 2019, **26**(5):399-401.

169. Afzal MF, Ghafoor S, Hamid MH: **Giardiasis in Children with Acute Watery Diarrhea**. *Annals of King Edward Medical University* 2019, **25**(4).

170. Bakarman MA, Hegazi MA, Butt NS: **Prevalence, characteristics, risk factors, and impact of intestinal parasitic infections on school children in Jeddah, Western Saudi Arabia**. *Journal of epidemiology and global health* 2019, **9**(1):81.

171. Hussein JN, Meerkhan AA: **The Incidence Of Intestinal Parasites Among Children In Hivi Pediatric Hospital, Duhok, Iraq**. *Science Journal of University of Zakho* 2019, **7**(1):1-4.

172. Yu F, Li D, Chang Y, Wu Y, Guo Z, Jia L, Xu J, Li J, Qi M, Wang R: **Molecular characterization of three intestinal protozoans in hospitalized children with different disease backgrounds in Zhengzhou, central China**. *Parasites & vectors* 2019, **12**(1):1-10.

173. Erzaiq ZS, Shaban MH, Adres M: **Relationship between gastroenteritis caused by Helicobacter pylori and Giardia lamblia in malnourished children**. *The Medical Journal of Tikrit University* 2020, **26**(2).

174. Bachtiar ZA, Hasanah APU, Yasin M, Isyaputri R, Budiono B, Basuki S: **The comparison of Giardia lamblia infection and nutritional status of elementary school students in Mandangin Island, Sampang and Mojo Village, Surabaya, Indonesia**. *Biomolecular and Health Science Journal* 2020, **3**(2):84-88.

175. Mohammed BA, Rasheed ZK, Jihad LJ, Abass KS: **Frequency of Giardia lamblia among Iraqi children in Kirkuk governorate**. *Systematic Reviews in Pharmacy* 2020, **11**(12):1909-1911.

176. Alshahethi MA, Edrees WH, Mogalli NM, Al-Halani AA, Al-Shehari WA, Reem A: **Distribution and risk factors for Giardia lamblia among children at Amran Governorate, Yemen**. *Pharmaceutical Research* 2020, **5**(3):34-37.

177. Teja SS, Swarna S, Jeyakumari D, Kanna V: **A study on intestinal parasitic infections among school children in Karaikal**. *Tropical Parasitology* 2020, **10**(2):79.

178. Abed ND: **Prevalence of Intestinal Parasites among Children in Khanaqin City East of Diyala/Iraq**. *Indian Journal of Public Health Research & Development* 2020, **11**(4).

179. Barati M, Taghipour A, Bakhshi B, Shams S, Pirestani M: **Prevalence of intestinal parasitic infections and Campylobacter spp. among children with gastrointestinal disorders in Tehran, Iran**. *Parasite Epidemiology and Control* 2021, **13**:e00207.

180. Neamah Ma: **Isolation and diagnosis of Entamoeba histolytica and Giardia lamblia from children infected with diarrheal at Mohammed AL-Moussawi Hospital in**. *public health* 2021, **6**(1).

181. Ashour AA, Ashour AA: **Epidemiological Study of Giardia Intestinalis parastie Among Children with Diarrhea in Duhok**. *Diyala Journal For Pure Science* 2021, **17**(01).

182. Hu Z, Zhong Y, Li J, Li Y: **Analysis on the detection rate of Giardia lamblia in children of Sichuan province of China**. 2022.
